# A Novel Flame-Retardant, Smoke-Suppressing, and Superhydrophobic Transparent Bamboo

**DOI:** 10.34133/research.0317

**Published:** 2024-02-14

**Authors:** Jiahui Su, Yadong Yang, Caichao Wan, Xingong Li, Yaling Chai, Huayun Chai, Jianzhong Yuan, Yiqiang Wu

**Affiliations:** ^1^ College of Materials Science and Engineering, Central South University of Forestry and Technology, Changsha 410004, P. R. China.; ^2^ Yihua Lifestyle Technology Co., Ltd., Huaidong Industrial Zone, Lianxia Town, Chenghai District, Shantou 515834, P. R. China.

## Abstract

Silica glass, known for its brittleness, weight, and non-biodegradable nature, faces challenges in finding suitable alternatives. Transparent wood, made by infusing polymers into wood, shows promise but is hindered by limited availability of wood in China and fire risks associated with its use. This study explores the potential of utilizing bamboo, which has a shorter growth cycle, as a valuable resource for developing flame-retardant, smoke-suppressing, and superhydrophobic transparent bamboo. A 3-layered flame-retardant barrier, composed of a top silane layer, an intermediate layer of SiO_2_ formed through hydrolysis-condensation of Na_2_SiO_3_ on the surface, and an inner layer of Na_2_SiO_3_, has been confirmed to be effective in reducing heat release, slowing flame spread, and inhibiting the release of combustible volatiles, toxic smoke, and CO. Compared to natural bamboo and other congeneric transparent products, the transparent bamboo displays remarkable superiority, with the majority of parameters being notably lower by an entire order of magnitude. It achieves a long ignition time of 116 s, low total heat release (0.7 MJ/m^2^), low total smoke production (0.063 m^2^), and low peak CO concentration (0.008 kg/kg). Moreover, when used as a substrate for perovskite solar cells, the transparent bamboo displays the potential to act as a light management layer, leading to a marked efficiency enhancement of 15.29%. The excellent features of transparent bamboo make it an enticing choice for future advancements in flame-retardant glasses and optical devices.

## Introduction

Silica glass, a widely used transparent material in the construction industry, has seen increased adoption as an essential building material over the past 50 years (Fig. S1). Its versatility is reflected in the global glass production reaching approximately 130 million tons in 2020 [1]. Despite its advantages such as high transparency, chemical inertness, and availability of raw materials, traditional silica glass still faces challenges including brittleness with a reported toughness of only 0.003 MJ m^−3^, high density, and substantial CO_2_ emissions and greenhouse gases during the manufacturing process [2–4]. Additionally, the non-biodegradable character of glass waste poses an important environmental concern on a global scale [5]. Spontaneous decomposition of waste inorganic glass in the environment typically takes millions of years [6]. Consequently, the development of novel and eco-friendly transparent materials for future use in sustainable construction has become imperative.

In recent years, there has been a surge of interest in transparent wood products, thanks to their remarkable features like high transparency, excellent mechanical strength, and superior thermal insulation properties [7–10]. This groundbreaking material is created by either removing or modifying the light-absorbing compositions of wood and then infusing the template with polymers that have refractive indices matching that of wood. Widely used polymers for this purpose include epoxy resin (EP, *n* ≈ 1.5), polyvinylpyrrolidone (PVP, *n* ≈ 1.53), and polymethyl methacrylate (PMMA, *n* ≈ 1.49). Transparent wood not only offers environmental benefits but also holds huge potential as a viable alternative to traditional glass materials. Nevertheless, there are several limitations associated with the utilization of transparent wood: (a) global wood scarcity, particularly in China, poses a challenge; despite efforts to increase production through plantations, it is projected that the demand for industrial roundwood in 2050 will exceed supply [11]; (b) the use of polymers in transparent wood makes it highly susceptible to fire, posing a potential hazard; and (c) there is a need to further enhance the functional properties of transparent wood beyond its basic optical and mechanical attributes.

Bamboo, often referred to as “the second forest”, boasts a rapid growth and regeneration rate, allowing it to reach maturity and be utilized as a building material within 4 to 7 years of growth [12]. With an output 4 times higher than wood per acre, bamboo is recognized for its exceptional efficiency. In terms of chemical composition, bamboo shares similarities with wood, mainly consisting of lignin, cellulose, and hemicellulose. Furthermore, the internal hierarchical structure of bamboo closely resembles that of wood, featuring high porosity and permeability because of neatly arranged vertical channels. This characteristic suggests the potential use of bamboo in the production of transparent composite materials. Transparent bamboo offers 3 distinct advantages over traditional silica glass. Firstly, the abundant and renewable nature of bamboo feedstock aligns with environmental sustainability goals. Secondly, transparent bamboo exhibits high light transmittance and haze, enabling privacy while facilitating the entry of natural light indoors [13,14]. Lastly, the low density and excellent ability to regulate temperature and humidity from a bamboo template further position it as a promising alternative to conventional glass. Transparent bamboo has been explored in a limited number of studies [15,16], and its transparency is attributed to impregnated organic polymers. Among the various types of organic polymers, EP and PMMA are the most commonly used, owing to their superior optical and mechanical properties. However, these polymers can release substantial heat and toxic smoke during burning, presenting a notable fire hazard and endangering human safety. Consequently, a big challenge lies in developing transparent bamboo composites capable of simultaneously delivering exceptional optical properties and flame retardancy.

Taking these concerns into account, in this study, we, for the first time, develop a novel flame-retardant transparent bamboo material, achieved by impregnating an inorganic liquid sodium silicate (Na_2_O·nSiO_2_) into the delignified bamboo structure using a facile and efficient vacuum-impregnation technique. Subsequently, a hydrophobic treatment is applied to the intermediate product. The resulting transparent bamboo shows notable features, such as reduced heat, smoke, and CO release attributed to the 3-layered flame-retardant barrier (comprising a top silane layer, an intermediate layer of SiO_2_ formed through hydrolysis–condensation of Na_2_SiO_3_ on the surface, and an inner layer of Na_2_SiO_3_), along with superhydrophobicity, boasting a contact angle reaching up to 154.7°. Additionally, it showcases outstanding optical properties, including high light transmittance of 71.6% and an impressive fog value of 96.7%. These characteristics enable it to function as a light management layer, remarkably enhancing overall power conversion efficiency (PCE) by 15.29% when used as a substrate for perovskite solar cells (PSCs).

## Results and Discussion

### Construction of flame-retardant, smoke-suppressing, and superhydrophobic transparent bamboo

**Fig. 1.**
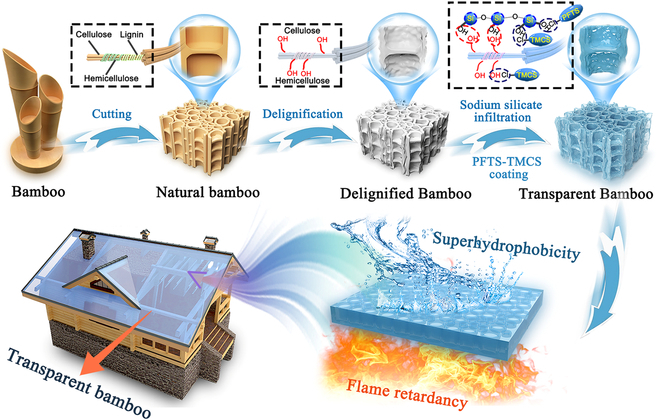
Schematic illustration of the preparation method for flame-retardant, smoke-suppressing, and superhydrophobic transparent bamboo.

Figure 1 presents a schematic diagram illustrating the preparation strategy for the flame-retardant, smoke-suppressing, and superhydrophobic transparent bamboo. The characteristic color of bamboo stems from lignin’s light absorption capacity, which accounts for approximately 80% to 95% of light absorption, while cellulose and hemicellulose are optically colorless and do not contribute to bamboo’s opaque nature. Consequently, delignification becomes a crucial step in achieving transparent bamboo. Additionally, bamboo’s dense vascular bundle tissue and glial substances pose a challenge for modification reagents to penetrate effectively. Research has shown that removing lignin enhances bamboo’s porosity and permeability [17]. Therefore, after delignification, vacuum impregnation is used to introduce liquid sodium silicate into the bamboo’s interior, resulting in flame-retardant transparent bamboo with high light transmittance. Notably, liquid sodium silicate serves as a commonly used inorganic intumescent material, forming a solid foamy layer against flames and smokes. Finally, a dip-coating method is used to graft the mixed reagent of perfluorooctyl trichlorosilane (PFTS) and trimethylchlorosilane (TMCS) onto the surface of the flame-retardant transparent bamboo samples [18–20], thereby imparting it with exceptional hydrophobic properties.

### Chemical compositions and crystal structure of transparent bamboo

**Fig. 2.**
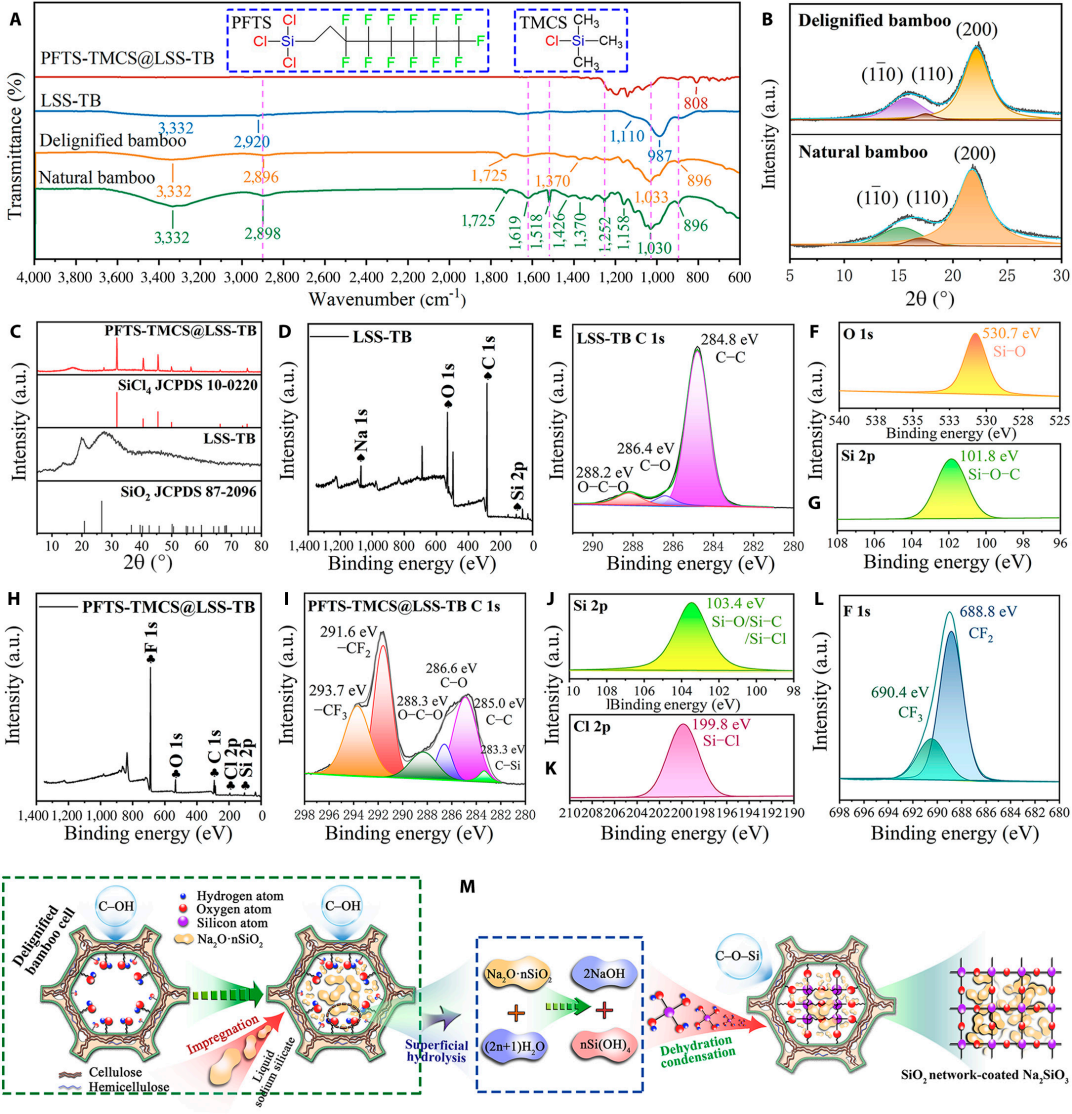
Chemical compositions and crystal structures of the natural bamboo and transparent bamboo. (A) FTIR spectra with insets showing the chemical structure of PFTS and TMCS. (B) XRD patterns of the natural bamboo and delignified bamboo. (C) XRD patterns of LSS-TB and PFTS-TMCS@LSS-TB. (D to G) XPS spectra of LSS-TB: (D) survey spectrum, (E) C 1s spectrum, (F) O 1s spectrum, and (G) Si 2p spectrum. (H to L) XPS spectra of PFTS-TMCS@LSS-TB: (H) survey spectrum, (I) C 1s spectrum, (J) Si 2p spectrum, (K) Cl 2p spectrum, and (L) F 1s spectrum. (M) Schematic diagram illustrating the potential mechanism of liquid sodium silicate impregnation into the delignified bamboo cell.

The chemical compositions of bamboo are analyzed through Fourier transform infrared spectroscopy (FTIR) before and after the delignification, penetration of liquid sodium silicate, and PFTS-TMCS coating processes. As depicted in Fig. 2A, the characteristic absorption bands of natural bamboo primarily originate from its 3 main components: cellulose, hemicellulose, and lignin. The band at 3,332 cm^−1^ is ascribed to the stretching vibrations of hydroxyl groups (O–H), while the band at 2,898 cm^−1^ corresponds to the methylene groups’ (CH–H) stretching vibrations [21]. Additionally, the band at 1,725 cm^−1^ is associated with the tensile vibration of acetyl groups in hemicellulose [22], while the tensile vibrations of C=C and C–O groups on the aromatic ring of lignin produce the bands at approximately 1,619 cm^−1^ and 1,252 cm^−1^, respectively [23]. Moreover, the absorption band at 1,518 cm^−1^ corresponds to the tensile vibration of the lignin aromatic skeleton, and the band at 1,370 cm^−1^ is related to the C–H bending vibration in cellulose and hemicellulose [24]. The bands at 1,426, 1,158, 1,033, and 896 cm^−1^ are assigned to the (C_6_)–CH_2_ bending, C–O–C pyranose ring skeletal vibration, C–O–C stretching vibration, and β-glycosidic linkages between the sugar units, which are characteristic features of cellulose in FTIR spectra [25].

In the FTIR spectrum of the delignified bamboo, the characteristic bands of lignin at 1,619, 1,518, and 1,252 cm^−1^ decrease dramatically, indicating the removal of most lignin. Furthermore, in the FTIR spectrum of liquid sodium silicate-transparent bamboo (LSS-TB), new characteristic bands appear, signifying the addition of new functional groups. For instance, the characteristic band at 987 cm^−1^ corresponds to the tensile vibration of Si–O bonds, while the shoulder band at 1,110 cm^−1^ is associated with the symmetrical shrinkage of Si–O–Si [26], indicating the penetration of liquid sodium silicate into the interior of delignified bamboo. Furthermore, the FTIR analysis shows that the band at 3,332 cm^−1^ becomes broader, revealing a decrease in the number of free hydroxyl groups in bamboo and an increase in the number of associated hydroxyl groups [27]. This phenomenon is attributed to the reaction between the –OH groups of bamboo and liquid sodium silicate, forming Si–O–C bonds. Following the modification of PFTS-TMCS, a new absorption band at 808 cm^−1^ is observed, which corresponds to the Si–(CH_3_)_3_ hydrophobic groups [28], demonstrating the successful construction of a hydrophobic layer on the surface of the transparent bamboo. The FTIR bands and their assignments are summarized in Table S1.

X-ray diffraction (XRD) analysis is employed to investigate the crystal structure. The XRD pattern of the delignified bamboo is identical to that of the natural bamboo. Figure 2B displays 3 characteristic peaks at 15.3°, 17.0°, and 21.8°, which are attributed to the crystal planes of (1$\bar{1}$0), (110), and (200) of cellulose Iβ. This allomorph is the primary crystalline form of cellulose found in higher plants [29]. It is evident that the delignification process did not alter the lattice structure of cellulose. However, it did increase the crystallinity index from 55.4% to 73.7% by eliminating amorphous lignin, which was tested by the Segal method [30]. For LSS-TB, there are 2 new diffraction peaks at 2*θ* = 20.8° and 26.6° (Fig. 2C), which are well indexed to the (100) and (110) planes of the standard Joint Committee on Powder Diffraction Standards (JCPDS) card no. 87-2096 for SiO_2_ (low-temperature quartz), respectively. The possible explanation for the presence of SiO_2_ is that the liquid sodium silicate on the surface of the delignified bamboo undergoes hydrolysis with the internal water, resulting in the formation of silicic acid. Subsequently, the Si–OH bond of the silicic acid undergoes dehydration condensation with moisture or free hydroxyl groups present on the bamboo cell wall, thereby forming Si–O–Si or Si–O–C structures (as depicted in Fig. 2M) [31]. Following the PFTS-TMCS modification, the XRD pattern of PFTS-TMCS@LSS-TB displays the characteristic Cl–Si structure, which is proved by the standard JCPDS card no. 10-0220 for SiCl_4_ (Fig. 2C).

X-ray photoelectron spectroscopy (XPS) analysis is utilized to further investigate the chemical functional groups of LSS-TB and PFTS-TMCS@LSS-TB. The XPS survey spectrum of LSS-TB (Fig. 2D) reveals the detection of 4 main elements (Si, C, O, and Na) at 103.1, 284.8, 531.1, and 1,071.1 eV, respectively. In the high-resolution XPS spectrum of C 1s for LSS-TB (Fig. 2E), 3 fitting peaks are observed at 284.8, 286.4, and 288.2 eV, corresponding to C–C, C–O, and O–C–O, respectively. The high-resolution XPS spectra of O 1s and Si 2p for LSS-TB (Fig. 2F and G) exhibit 2 peaks at 530.7 and 101.8 eV, which are attributed to the Si–O and Si–O–C groups [32], respectively. These findings further confirm the penetration of liquid sodium silicate into the interior of delignified bamboo.

The XPS survey spectrum of PFTS-TMCS@LSS-TB is depicted in Fig. 2H, revealing a prominent signal of the F element while the Na signal diminishes. This observation indicates that the surface of LSS-TB is enveloped with the PFTS-TMCS reagent. In Fig. 2I, the high-resolution XPS spectrum of C 1s for PFTS-TMCS@LSS-TB displays 6 fitting peaks at 283.3, 285.0, 286.6, 288.3, 291.6, and 293.7 eV, corresponding to C–Si, C–C, C–O, O–C–O, –CF_2_, and –CF_3_ [33], respectively. Additionally, the peak in the high-resolution XPS spectrum of Si 2p appears at 103.4 eV (Fig. 2J), attributed to the Si–O/Si–C/Si–Cl [34]. Furthermore, the presence of the Si–Cl bond in the XPS Cl 2p spectrum (Fig. 2K) of PFTS-TMCS@LSS-TB proves the grafting of chlorosilane onto the surface of transparent bamboo. The high-resolution F 1s spectrum (Fig. 2L) presents 2 fitting peaks at 690.4 and 688.8 eV, associated with the CF_3_ and CF_2_ moieties from PFTS [35], respectively. A plausible reaction mechanism between PFTS-TMCS and LSS-TB is illustrated in Fig. S2.

### Microstructure and thermal stability of transparent bamboo

**Fig. 3.**
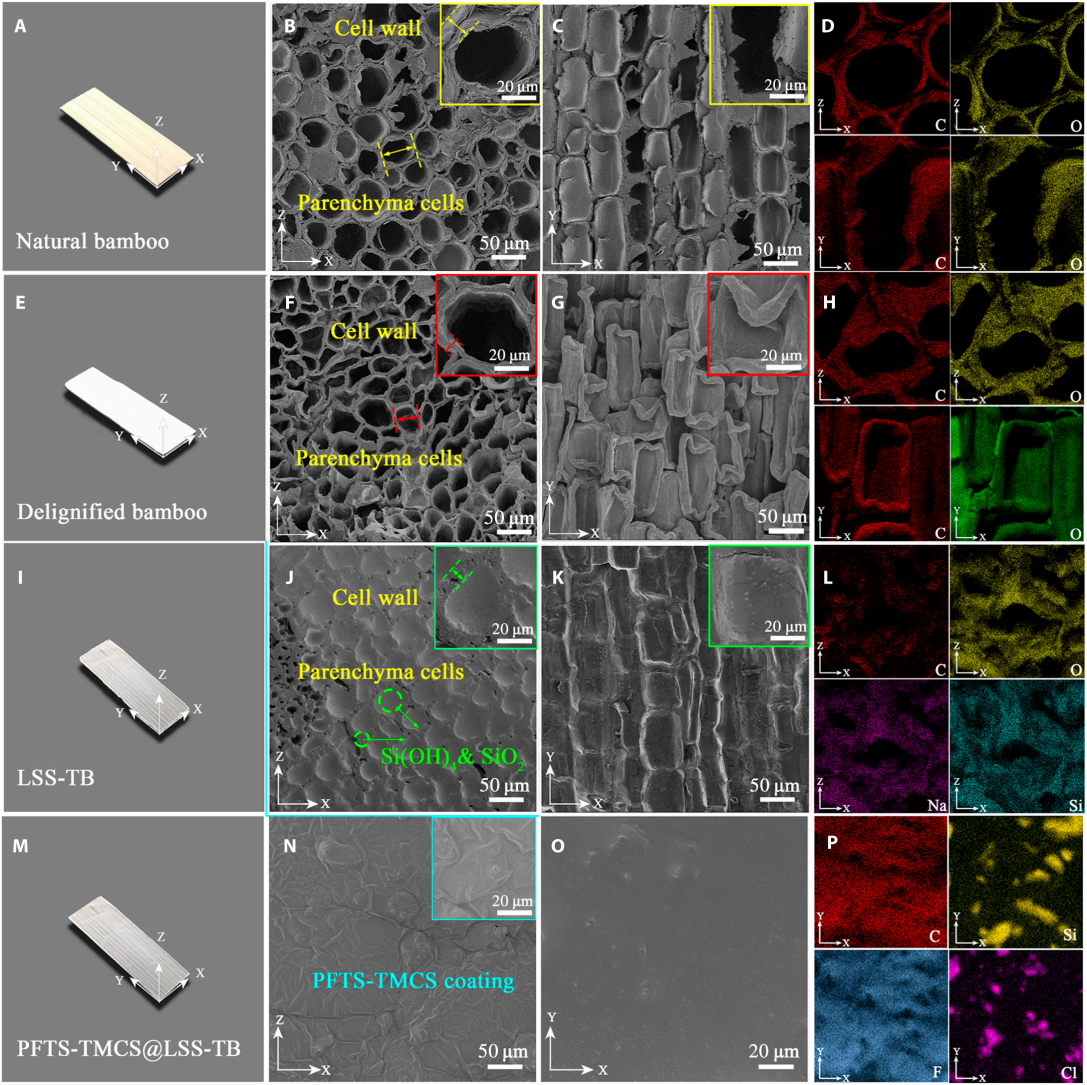
Structural characterizations of the natural bamboo, delignified bamboo, LSS-TB, and PFTS-TMCS@LSS-TB. (A) Photograph of the natural bamboo. (B and C) SEM images of the natural bamboo: (B) cross-sectional image showing the bamboo lumen structure (in the *XZ* plane); (C) longitudinal image showing the lumina along the growth direction (in the *XY* plane). (D) EDX mapping patterns of the natural bamboo. (E) Photograph of the delignified bamboo. (F and G) SEM images of the delignified bamboo: (F) cross-sectional image; (G) longitudinal image. (H) EDX mapping patterns of the delignified bamboo. (I) Photograph of LSS-TB. (J and K) SEM images of LSS-TB: (J) cross-sectional image; (K) longitudinal image. (L) EDX mapping patterns of LSS-TB. (M) Photograph of PFTS-TMCS@LSS-TB. (N and O) SEM images of PFTS-TMCS@LSS-TB: (N) cross-sectional image; (O) longitudinal image. (P) EDX mapping patterns of PFTS-TMCS@LSS-TB.

The use of scanning electron microscopy (SEM) provides insights into the morphology and structure of both natural and transparent bamboo, as depicted in Fig. 3. The images in Fig. 3A, E, I, and M illustrate the visual appearance of natural bamboo, delignified bamboo, LSS-TB, and PFTS-TMCS@LSS-TB, respectively, indicating the complete transformation of yellow natural bamboo into a white material after the delignification. When observed from a distance, both LSS-TB and PFTS-TMCS@LSS-TB exhibit a subtle translucency due to their high haze characteristic, which will be further elaborated upon in the next section. The SEM images of the natural bamboo (Fig. 3B and C) reveal closely arranged thin-walled parenchyma cells with an irregular shape, each possessing a diameter of around 50 μm along the direction of tree growth. Following delignification, the 3-dimensional anisotropic porous structure remains intact, with a slight shrinkage observed on the cell wall. This shrinkage indicates a reduction in cell wall stiffness, thereby facilitating the penetration of impregnating agents (Fig. 3F and G).

Figure 3J and K show SEM images of LSS-TB, clearly illustrating that the gaps and pores of bamboo parenchyma cells are entirely filled, demonstrating the effective penetration of liquid sodium silicate into the inner regions of delignified bamboo. This is further supported by the uniform distribution of the Si element in the energy dispersive x-ray (EDX) mapping pattern (Fig. 3D, H, and L), which aligns with the findings from FTIR, XPS, and XRD analyses, confirming the successful penetration of liquid sodium silicate. The incorporation of liquid sodium silicate enhances the flame retardancy of delignified bamboo framework. Subsequent to the hydrophobic treatment with PFTS and TMCS, the filled lumina become indiscernible, replaced by a thin coating layer (Fig. 3N and O). Furthermore, the EDX mapping pattern of PFTS-TMCS@LSS-TB indicates the homogeneous distribution of Cl and F elements (Fig. 3P) on the surface of LSS-TB.

We performed thermal gravimetric (TG) and differential TG (DTG) analyses to evaluate the thermal stability of natural bamboo and PFTS-TMCS@LSS-TB. As depicted in Fig. S3, the pyrolysis process of natural bamboo unfolds in 3 stages [36]: (a) The first stage, occurring at temperatures below 200 °C, results in a 6% weight loss mainly attributed to the evaporation of adsorbed water. (b) In the second stage, spanning from 200 to 450 °C, cellulose shows a sharp pyrolysis peak at 317 °C, while the shoulder peak at 293 °C corresponds to the thermal decomposition of hemicellulose. This stage releases a substantial number of combustible substances (CH_4_, wood tar, etc.) [37], leading to rapid and substantial weight loss and flame burning. Subsequently, under oxidative conditions, charcoal calcination occurs between 400 and 450 °C, replacing the flame-burning process and resulting in a distinct exothermic peak at 439 °C in the DTG curve [38]. (c) The third stage, ranging from 450 to 800 °C, is characterized by weight stabilization and the formation of a stable structure of carbon slag.

In the case of PFTS-TMCS@LSS-TB, the initial weight loss below 200 °C is primarily attributed to the evaporation of adsorbed water. Furthermore, within the temperature range of 200 to 450 °C, PFTS-TMCS@LSS-TB exhibits a mere 17.5% weight loss, distinctly lower than that of natural bamboo (90.78%) in the same range (Fig. S3A). Additionally, the maximum weight loss rate markedly decreases from 57.3% min^−1^ to 4.4% min^−1^ (Fig. S3B), indicating a strong inhibition of the pyrolysis reaction at this stage.

### Flame-retardant, smoke-suppressing, and CO release properties of transparent bamboo

In order to assess the combustion properties of transparent bamboo, cone calorimeter (CONE) tests were conducted at a radiation intensity of 50 kW m^−2^ following ISO5660-2 guidelines. The summarized results are available in Table S2. The ignition time (TTI) refers to the duration from exposure to heat radiation until continuous surface ignition occurs under a specific heat flux radiation intensity. It is clear from the data that natural bamboo has a distinctly shorter TTI (20 s) compared to PFTS-TMCS@LSS-TB (116 s), confirming a higher susceptibility to fire and an increased risk of burning associated with natural bamboo. Nevertheless, by incorporating liquid sodium silicate and PFTS-TMCS into the structure, the TTI is increased by 96 s. This extended TTI translates to an escape distance of 576 m based on the average person’s standard running pace of 6 m s^−1^, thus reducing potential casualties. Therefore, a larger TTI value indicates a more superior flame retardancy property.

**Fig. 4.**
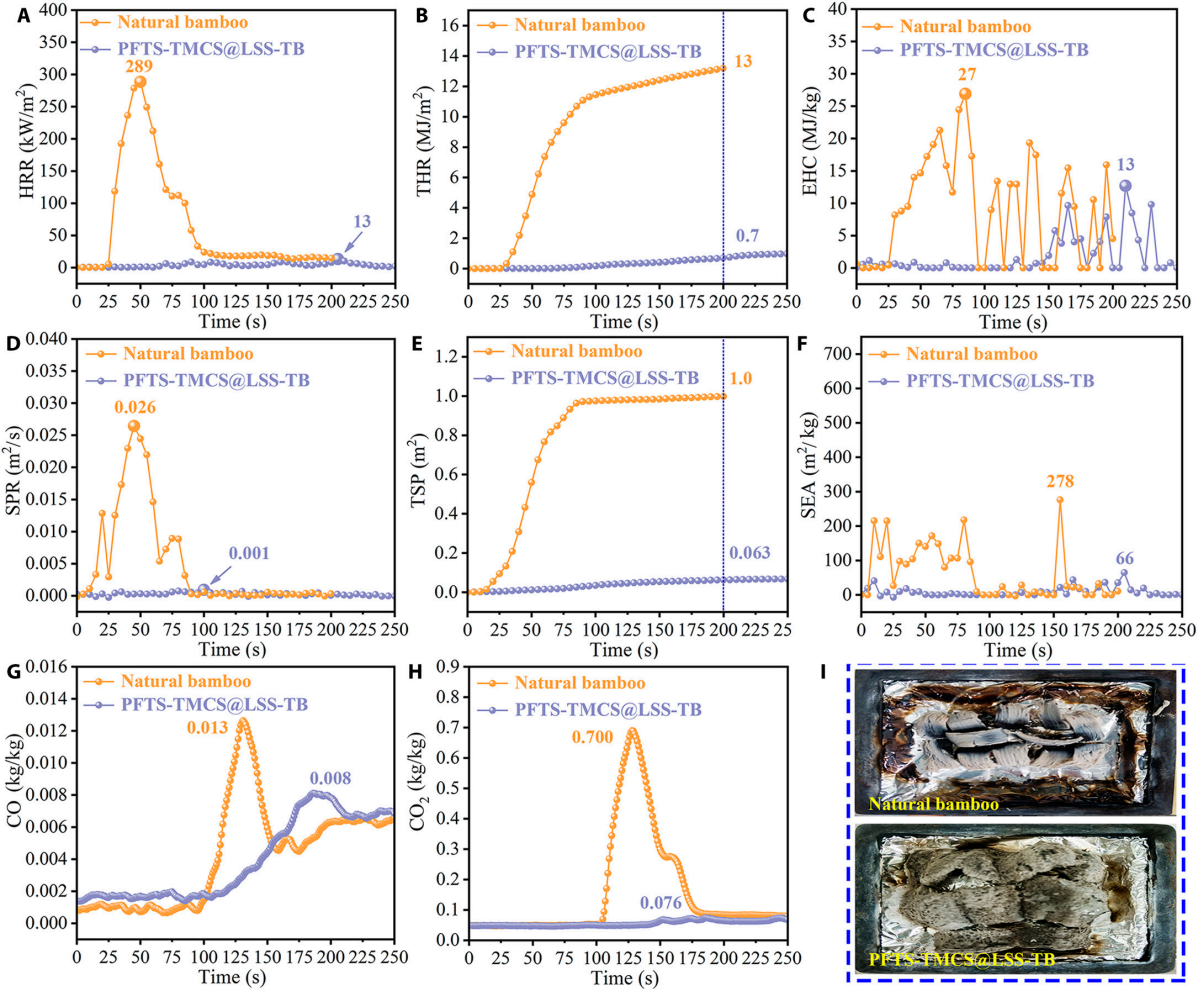
Combustion properties of the natural bamboo and PFTS-TMCS@LSS-TB. (A) HRR. (B) THR. (C) EHC. (D) SPR. (E) TSP. (F) SEA. (G) Release of CO concentration. (H) Release of CO_2_ concentration. (I) Digital photos of post-combustion morphologies.

The heat release rate (HRR), also known as fire intensity, indicates the rate of heat released per unit area of the sample after ignition under the heat radiation and heat flow intensity of a preset heater. The spread of fire is directly affected by HRR, and reducing this rate can extend the time available for rescue efforts and effectively restrain the growth of the fire [39]. As depicted in Fig. 4A, natural bamboo exhibits a rapid increase in the HRR plot, reaching its initial exothermic peak at 50 s due to the formation and subsequent combustion of an intumescent char layer [40]. The second exothermic peak at 81 s is a result of the swift combustion of flammable gaseous products. However, upon the introduction of liquid sodium silicate and PFTS-TMCS, a noticeable reduction in both the peak of HRR (PHRR) and mean HRR (MHRR) values is observed. Specifically, natural bamboo demonstrates high PHRR and MHRR values of 289 and 73 kW m^−2^, respectively, whereas PFTS-TMCS@LSS-TB displays considerably lower values of 13 and 3.9 kW m^−2^, respectively (approximately 1/20). Moreover, the HRR curve of PFTS-TMCS@LSS-TB tends toward the horizontal axis (*y* = 0), indicating an extremely low rate of heat release.

The total heat release (THR) reflects the cumulative heat released by materials during combustion, and a higher THR reflects a greater risk of material ignition. Consequently, reducing the total amount of material is advantageous in postponing combustion. As depicted in Fig. 4B, natural bamboo exhibits a THR of 13 MJ m^−2^ at 200 s, which is 18.6 times higher than that of PFTS-TMCS@LSS-TB (0.7 MJ m^−2^). The lower TTI, HRR, and THR of PFTS-TMCS@LSS-TB are attributed to the presence of superficial grating silane molecules, which act as the primary fire barrier, effectively enhancing flame-retardant capability [41,42]. Additionally, the interlayered SiO_2_ network functions as a secondary physical barrier due to its high melting point and low thermal conductivity. Finally, the internal sodium silicate, a widely used intumescent inorganic material, acts as a third barrier by forming a solid foamy layer on the bamboo skeleton’s surface to resist the flame (refer to Fig. S4 for the SEM image of PFTS-TMCS@LSS-TB after combustion) [43]. Moreover, a method to prevent the thermal decomposition of liquid sodium silicate via oxide layer deposition, facilitated by the interaction with atmospheric gases, has been proposed [44]. This trilaminar structure effectively delays the spread of the flame. For convenience, videos of the combustion tests for both natural bamboo and PFTS-TMCS@LSS-TB are included in the Supplementary Materials (Movies S1 and S2).

The effective heat of combustion (EHC) signifies the heat release from the combustible volatiles formed from material pyrolysis in the flame, providing a comprehensive insight into the flame-retardant mechanism of transparent bamboo. As illustrated in Fig. 4C, the EHC of PFTS-TMCS@LSS-TB is consistently lower than that of natural bamboo. Specifically, the PEHC of PFTS-TMCS@LSS-TB is recorded at 13 MJ kg^−1^ at 209 s, whereas that of the bamboo feedstock is 27 MJ kg^−1^ at 85 s. This discrepancy suggests that PFTS-TMCS@LSS-TB generates fewer combustible volatiles during heat degradation. Thus, it can be inferred that the incorporation of liquid sodium silicate and PFTS-TMCS restrains the release of combustible volatiles during the heat degradation of bamboo.

Previous studies have indicated that the majority of fire-related fatalities result from the inhalation of toxic smoke. To assess the efficacy of PFTS-TMCS@LSS-TB in mitigating smoke emissions during combustion, we examined the smoke production rate (SPR), total smoke production (TSP), and mean specific extinction area (MSEA). SPR and TSP are pivotal parameters influencing the performance of insulating materials. As depicted in Fig. 4D, the SPR profiles during combustion are presented. Natural bamboo exhibits an initial release of smoke at the onset of combustion, with a gradual increase in SPR as the heating duration progresses. The peak SPR (PSPR) of natural bamboo is 0.026 m^2^ s^−1^ at around 45 s, followed by a gradual decline. A third peak emerges at roughly 77 s, after which the SPR rapidly diminishes to 0. In contrast, the SPR curve of PFTS-TMCS@LSS-TB nearly aligns with the *X*-axis (*y* = 0), indicating a markedly reduced rate of smoke production during combustion. This can be attributed to the formation of an insulating foamy glassy layer on the surface of the bamboo structure, induced by the expansion of liquid sodium silicate at high temperatures coupled with a reduction in density [45]. Consequently, the encapsulation of this layer, along with the SiO_2_ network layer, effectively restrains the release of volatiles, thereby reducing the SPR. Furthermore, the SPR curves are closely correlated with the HRR curves (Fig. 4A) of the samples, confirming that the quantity of smoke released during combustion is proportional to the HRR. The TSP curves in Fig. 4E represent the total smoke accumulation when a unit sample area is combusted. In contrast to PFTS-TMCS@LSS-TB (0.063 m^2^), the TSP of natural bamboo (1.0 m^2^) is 14.9 times greater. Moreover, the MSEA value, which indicates the smoke production capacity per unit mass of the burning mixture, decreases from 110 to 8.6 m^2^ kg^−1^ after the incorporation of liquid sodium silicate and PFTS-TMCS, as illustrated in Fig. 4F. Consequently, fewer smokes are produced throughout the combustion process of PFTS-TMCS@LSS-TB. This is attributed to the flame-retarding and smoke-suppressing capabilities of liquid sodium silicate and PFTS-TMCS.

The inhalation of flammable CO can pose a severe suffocation risk. Alarming statistics highlight that CO is a leading factor in poisoning deaths resulting from fire-related incidents [46], emphasizing the urgent need to investigate the CO emission levels during combustion. Figure 4G and H display the CO and CO_2_ concentration curves for natural bamboo and PFTS-TMCS@LSS-TB. Notably, the peak CO concentration of natural bamboo occurs at 131 s, earlier than PFTS-TMCS@LSS-TB at 187 s. Additionally, the peak CO yield (PCOY) of natural bamboo decreases by 38.5% upon the addition of liquid sodium silicate and PFTS-TMCS, indicating a delayed CO release and a prominent reduction in absolute CO quantity. In Fig. 4H, it is evident that the peak CO_2_ yield (PCO_2_Y) of PFTS-TMCS@LSS-TB is 0.076 kg/kg, just 1/9th of that of natural bamboo (PCO_2_Y = 0.700 kg/kg). The observed decrease in CO and CO_2_ release in the PFTS-TMCS@LSS-TB sample is attributed to the formation of a dense foamy layer, which acts as a physical barrier impeding gas release. Furthermore, any unreacted sodium silicate in the sample can react with generated CO_2_ (Na_2_SiO_3_ + CO_2_ + H_2_O = H_2_SiO_3_ + Na_2_CO_3_), serving as a CO_2_ reservoir during combustion. The lower CO_2_ content in the system results in reduced gas phase combustion reactions and decreased smoke release [47].

Figure 4I presents digital photos of the post-combustion morphologies. Notably, the combustion product of natural bamboo reveals a warped structure, suggesting the incineration of the bamboo framework. Conversely, upon the addition of liquid sodium silicate and PFTS-TMCS, the combustion product presents a substantially increased volume, with the surface adorned by abundant porous networks. These networks are ascribed to the expansion of liquid sodium silicate, while the produced SiO_2_ exhibits low heat conductivity, thereby augmenting the material’s thermal insulation and flame-retardant properties [48].

**Fig. 5.**
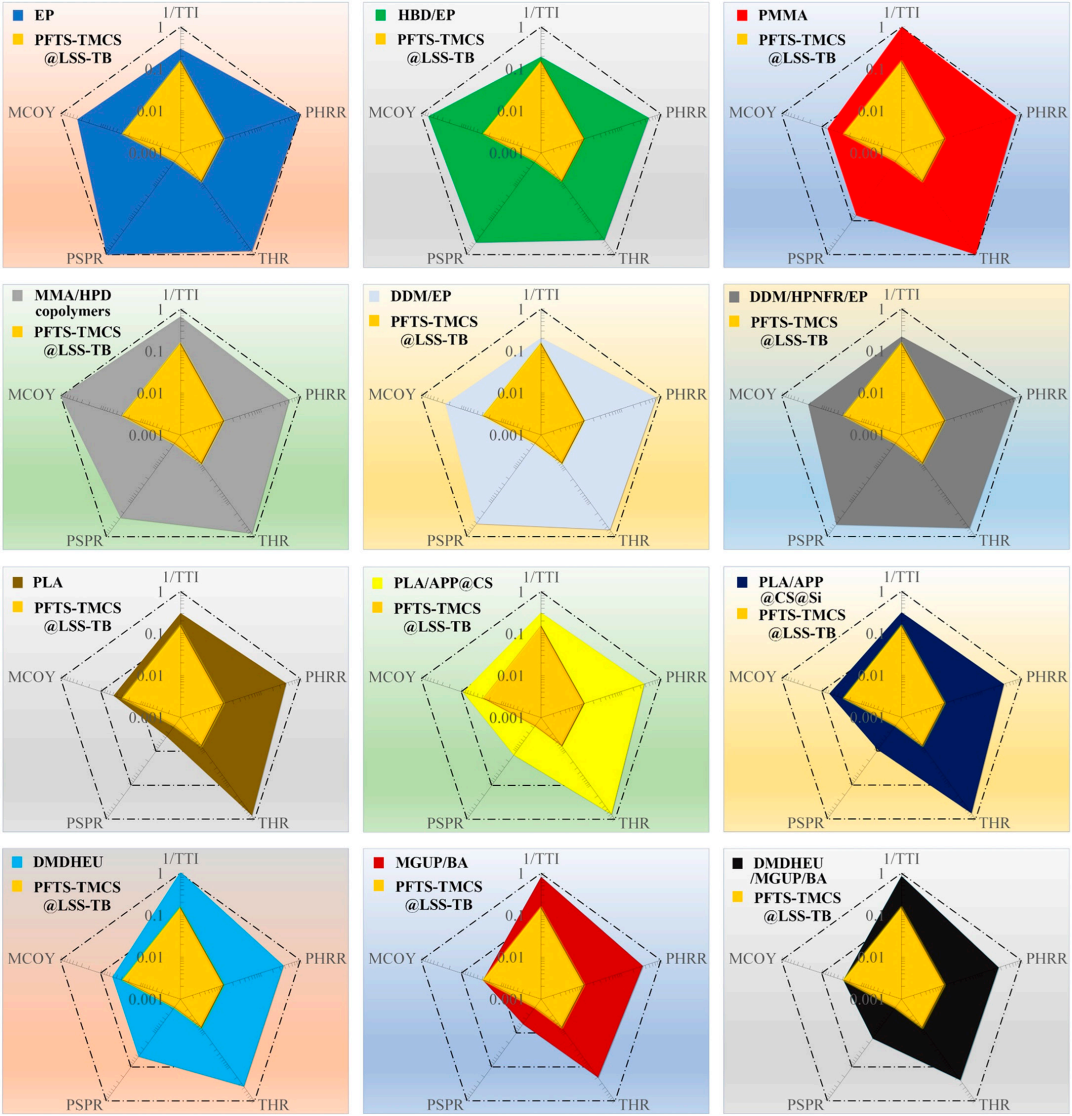
Comparative analysis of flame-retardant, smoke-suppressing, and CO release properties: PFTS-TMCS@LSS-TB vs. congeneric transparent materials.

Like transparent bamboo, several polymer-based materials, including EP [49], PMMA [50], and polylactic acid (PLA) [51], hold promise for novel glass-like applications. Many of these materials, or their corresponding composites, demonstrate commendable fire-resistant properties, for instance, N/P/S containing flame retardant (HBD)/EP [49], MMA/phosphorus-containing flame retardant (HPD) copolymers [50], PLA/ammonium polyphosphates (APP)@chitosan (CS) [51], PLA/APP@CS@Si [51], 4,4-diaminodiphenylmethane (DDM)/EP [52], DDM/hyperbranched P/N-containing flame retardant (HPNFR)/EP [52], 1,3-dimethylol-4,5-dihydroxyethyleneurea (DMDHEU) [53], methylolated guanylurea phosphate (MGUP)/boric acid (BA) [53], and DMDHEU/MGUP/BA [53]. Hence, they serve as reference points for transparent bamboo and enable a comparison of their flame-retardant properties. Figure 5 presents a comparative analysis of flame-retardant, smoke-suppressing, and CO release properties of PFTS-TMCS@LSS-TB with those of similar transparent materials. In Table S3, we assess 5 crucial parameters, including 1/TTI, PHRR, THR, TSP, and MCOY. To standardize the data and eliminate the influence of magnitude, all values in Fig. 5 are normalized on a scale of 0 to 1 by dividing each value by the maximum within the group. The size of each pentagon reflects the level of fire risk, with larger areas indicating higher risks. The results clearly illustrate that PFTS-TMCS@LSS-TB has a clearly smaller pentagonal area compared to other similar transparent materials, underscoring its superior fire safety and smoke suppression capabilities for indoor glass applications.

### Optical, superhydrophobic, and mechanical properties of transparent bamboo

**Fig. 6.**
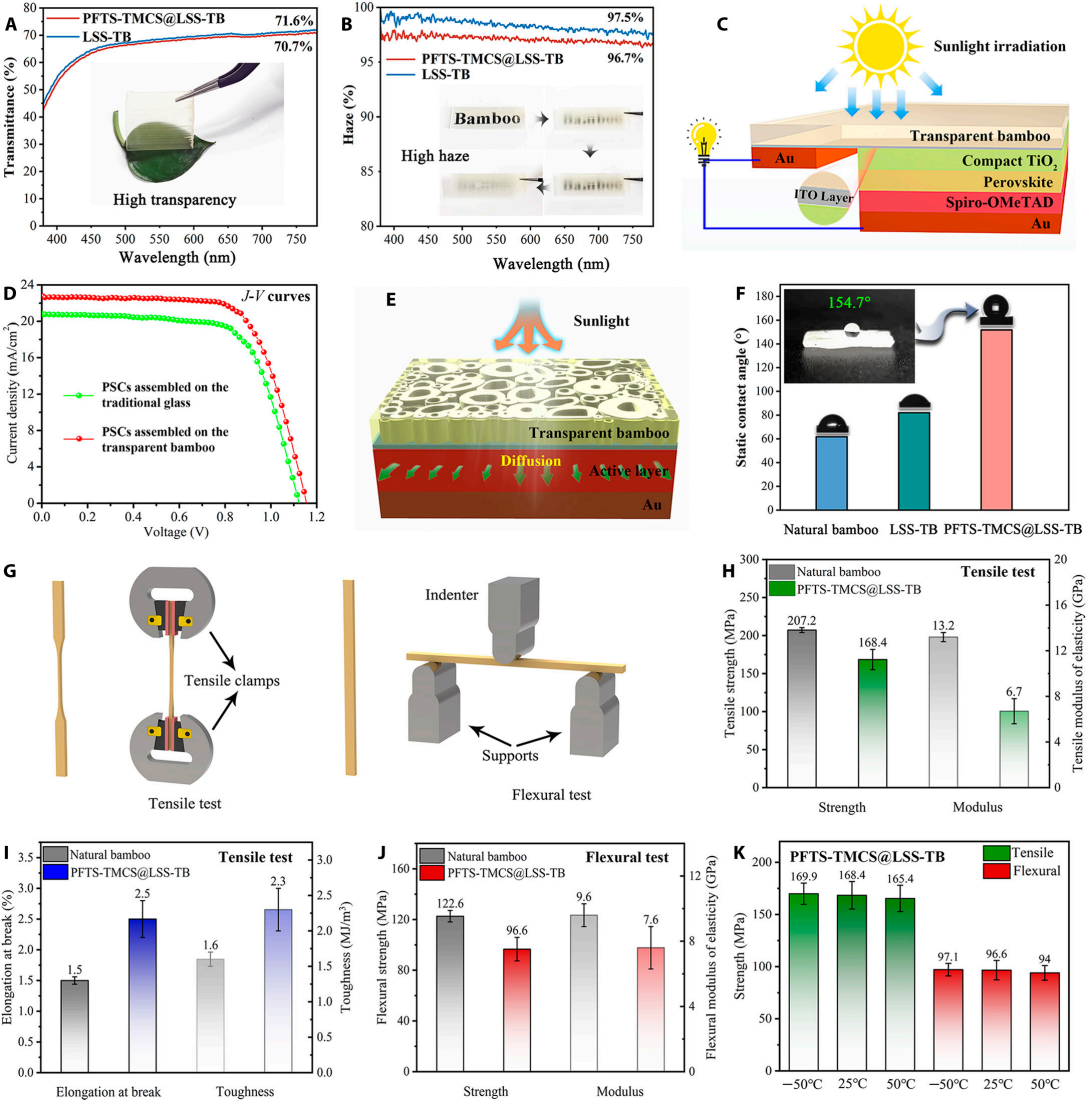
Optical, superhydrophobic, and mechanical properties of the transparent bamboo. (A) Transmittance spectra with an inset showing a piece of transparent bamboo placed on a leaf. (B) Haze spectra with insets demonstrating the gradual lifting of a piece of transparent bamboo to reveal its haze characteristic. (C) Schematic sketch showing the structure of PSCs assembled on the transparent bamboo substrate. (D) Current density–voltage properties of PSCs assembled on the transparent bamboo substrates or glass substrates. (E) Schematic of light distribution incident on a solar cell. (F) Static water contact angles with an inset showing a drop of water on the surface of the transparent bamboo. (G) Schematic diagram of the bamboo specimens for tensile and flexural tests. (H) Tensile strength and tensile modulus of elasticity. (I) Tensile elongation at break and toughness. (J) Flexural strength and flexural modulus. (K) Tensile and flexural strength at temperatures of –50, 25, or 50 °C

The primary reason for the lack of transparency in bamboo stems from the presence of porous lumen space at the core of the fiber and vessel cells, which have diameters in the range of tens of micrometers. Additionally, the yellow color of bamboo primarily arises from the high concentration of lignin, and the phenolic compounds in bamboo contain chromophoric groups [54]. To achieve transparency in bamboo, a delignification process is employed using NaClO_2_, followed by infiltration with a refractive-index-matched filler, liquid sodium silicate with a refractive index of 1.52. Figure 6A displays a photograph of a transparent bamboo sample that is 3.0 mm thick and positioned above a leaf, visually confirming the exceptional optical transparency of the material. In the visible range, both LSS-TB and PFTS-TMCS@LSS-TB demonstrate high optical transmittances of 70.7% and 71.6%, respectively. Furthermore, PFTS-TMCS@LSS-TB exhibits a high haze value of 96.7%, as depicted in Fig. 6B. At a distance of 3 cm, this high haze level gradually obscures the pattern of the background board, providing effective confidentiality protection. The integration of high transmittance and high haze makes our transparent bamboo a promising choice as an efficient light management layer for solar cells. PSCs have garnered considerable attention since their first discovery in 2009 because of high PCE [55]. The substrate plays a key role in PSCs as it not only determines the end use and functionality of the products but also impacts the overall sustainability of the final solar cell devices. Glass and plastics are broadly used as common substrates in solar cell applications. This study showcases the potential of using transparent bamboo as a viable alternative to traditional glass substrates for the assembly of PSCs. The structure and main components of PSCs are illustrated in Fig. 6C. The current density–voltage (*J*–*V*) curves of the PSCs assembled on the transparent bamboo substrate or glass substrate are presented in Fig. 6D. The photovoltaic properties of the PSCs, like short circuit density (*J*_SC_), open circuit voltage (*V*_OC_), fill factor (*FF*), and PCE, are derived from the *J*–*V* curves, as shown in Table S4. PSCs prepared on the traditional glass substrates have a PCE of 15.76% under 100 mW cm^−2^ AM 1.5G simulated irradiation, with a *J*_sc_ of 20.85 mA cm^−2^, a *V*_oc_ of 1.12 V, and an *FF* of 0.675. By utilizing the transparent bamboo substrate, a remarkable 15.29% enhancement is achieved in PCE (18.17%). Other parameters, i.e., *J*_sc_, *V*_oc_, and *FF*, also demonstrate higher values to 22.78 mA∙cm^−2^, 1.16 V, and 0.687, respectively. These enhancements are ascribed to 2 key factors: (a) the high transmittance of transparent bamboo, which enables light to reach the active layer with minimal loss, and (b) the highly diffuse nature of normal incident light upon reaching the top surface of the solar cell due to its high haze, resulting in an increase in the photon’s travel path within the PSCs and improving the likelihood of photon capture within the cell,s active region (Fig. 6E) [56].

Figure 6F depicts the average static water contact angle (*θ*) of 3 samples: natural bamboo, LSS-TB, and PFTS-TMCS@LSS-TB. The *θ* value allows for the classification of wetting behavior into 4 categories: superhydrophilic (0° < *θ* < 10°), hydrophilic (10° < *θ* < 90°), hydrophobic (90° < *θ* < 150°), and superhydrophobic (150° < *θ* < 180°). The natural bamboo and LSS-TB exhibit *θ* values of approximately 82° and 61.7°, respectively, indicating a hydrophilic surface dominated by the presence of numerous hydrophilic groups (e.g., C–OH and Si–OH). Notably, the *θ* value increases to 154.7° with the incorporation of PFTS and TMCS, successfully creating a superhydrophobic surface on the transparent bamboo. This surface shows potential for the development of self-cleaning window panes [57], allowing water droplets to effortlessly slide down the smooth, transparent bamboo surface and effectively collect dirt particles attached to it (refer to Fig. S5).

Figure 6G to I illustrate the typical tensile stress–strain tests for natural bamboo and PFTS-TMCS@LSS-TB. In the fiber direction, the natural bamboo exhibits a tensile strength of 207.2 ± 3.2 MPa and a tensile modulus of elasticity of 13.2 ± 0.4 GPa. In contrast, PFTS-TMCS@LSS-TB shows respective values of 168.4 ± 13.3 MPa and 6.7 ± 1.1 GPa (Fig. 6H). This difference is attributed to the removal of lignin, which disrupts the structural integrity of bamboo. Additionally, PFTS-TMCS@LSS-TB has a longitudinal elongation at break of approximately 2.5% ± 0.3%, representing a 66.7% increase compared to the natural bamboo’s value of 1.5% ± 0.06% (Fig. 6I). To calculate sample toughness, the tensile stress–strain curve is integrated. PFTS-TMCS@LSS-TB displays a toughness of 2.3 ± 0.3 MJ m^−3^, marking a 43.8% increase compared to the natural bamboo’s toughness of 1.6 ± 0.1 MJ m^−3^. Consequently, PFTS-TMCS@LSS-TB showcases enhanced tensile elongation at break and toughness but reduced tensile strength when compared with the natural bamboo. This behavior can be ascribed to the composition of natural bamboo, primarily consisting of stiff fiber bundles and lower-density, thin-walled tissue cells. The stiffness of the fiber bundles is primarily linked to their lignin content, so with lignin removal, the bamboo’s rigidity decreases while flexibility increases [58,59]. Additionally, the strong adhesion at the interface between bamboo fibers and sodium silicate enhances stress transfer efficiency among fibers, further contributing to the increased flexibility observed in PFTS-TMCS@LSS-TB. Moreover, Fig. 6J displays the flexural performances for the natural bamboo and PFTS-TMCS@LSS-TB. Similar to the tensile behavior, PFTS-TMCS@LSS-TB exhibits a decline in flexural strength and modulus. In the fiber direction, natural bamboo displays a flexural strength of 122.6 ± 4.5 MPa and a flexural modulus of 9.6 ± 0.7 GPa, whereas PFTS-TMCS@LSS-TB exhibits corresponding values of 96.6 ± 9.3 MPa and 7.6 ± 1.3 GPa. The flexural ductility of PFTS-TMCS@LSS-TB presents a substantial increase, as proved by a fracture deflection of 28.1 ± 5.5 mm, surpassing the natural bamboo’s fracture deflection of 10.9 ± 0.6 mm (Table S5) by a factor of 2.6. All the original data and corresponding stress–strain curves for these mechanical tests are available in Fig. S6. To further investigate the stability of the interface between delignified bamboo and LSS, especially in extreme environments, we conducted tensile and flexural stress–strain tests on PFTS-TMCS@LSS-TB specimens that were subjected to temperatures of –50, 25, or 50 °C for 24 h (Fig. S7). As shown in Fig. 6K, the tensile strengths at –50 °C and 50 °C are 169.9 ± 10.2 and 165.4 ± 12.6 MPa, respectively, which are quite close to the strength tested at room temperature (168.4 ± 13.3 MPa). Similarly, the differences in flexural strength at –50, 25, or 50 °C are also small (97.1 ± 5.9, 96.6 ± 9.3, and 94.0 ± 7.0 MPa). These results confirm the robustness and stability of the interface between delignified bamboo and LSS, even under extreme temperature conditions.

## Discussion

We create a new flame-retardant, smoke-suppressing, and superhydrophobic transparent bamboo composite, utilizing natural bamboo as a readily available and abundant raw material. Through delignification, the removal of lignin in bamboo, and subsequent impregnation with liquid sodium silicate and surface modification, we have achieved a transparent bamboo with 71.6% optical transmittance and 96.7% haze, making it ideal for uniform indoor lighting and privacy protection. Besides, these characteristics make it suitable for use as a light management layer, leading to a notable PCE improvement of 15.29% when utilized as a substrate for PSCs. Additionally, this transparent bamboo demonstrates superior thermal stability and exhibits outstanding flame-retardant and smoke-suppressing abilities, with a TTI delayed by 96 s and prominent decreases in PHRR, MHRR, THR, PEHC, MEHC, PSPR, TSP, MSEA, PCOY, and PCO_2_Y to around 1/22, 1/19, 1/19, 1/2, 1/8, 1/26, 1/16, 1/13, 1/1.6, and 1/9 of those of natural bamboo, respectively. Moreover, the transparent bamboo also displays superb superhydrophobicity, with a water contact angle as high as 154.7°. Therefore, this transparent bamboo is anticipated to serve as an innovative, eco-friendly, and self-cleaning material for buildings, as well as a promising option for optical applications in the future.

## Materials and Methods

### Materials

To prepare transparent bamboo, we sourced flattened bamboo boards (*Phyllostachys heterocycla*) directly from Zhejiang Dechang Bamboo-Wood Co., Ltd. in China as our raw material. Other chemicals, including sodium chlorite (NaClO_2_, 80%), glacial acetic acid (CH_3_COOH), absolute ethanol (C_2_H_5_OH), liquid sodium silicate (Na_2_O·nSiO_2_, *m* = 2.25), PFTS, and TMCS, were procured from Aladdin Reagent Co., Ltd. in Shanghai, China and used without further purification.

### Delignification of bamboo

To initiate the delignification process, the bamboo strips were cut into slices with a thickness of 0.3 cm, followed by meticulous washing with distilled water and absolute ethanol to remove any impurities. Subsequently, the chips were dried in an oven at 105 °C for 30 min. These prepared chips were then immersed in a 4% NaClO_2_ solution with a pH of 4.6 (adjusted using glacial acetic acid) and allowed to soak in a reactor at 85 °C for a duration of 2 h. After completion, the sample was taken out from the solution and rinsed 3 times with distilled water and absolute ethanol to eliminate any residual chemicals. Finally, the delignified bamboo was dried at 105 °C.

### Preparation of transparent bamboo

After delignification and drying, the delignified bamboo slices were placed at the bottom of a beaker and dipping them in a sodium silicate solution. Subsequently, the solution was degassed under a vacuum of 200 Pa to eliminate any gas from the bamboo slices. Following a period of 5 h, the vacuum was released, enabling the delignified bamboo slices to be filled with sodium silicate under atmospheric pressure. The resulting transparent bamboo sample, designated as LSS-TB, was then positioned on sand film paper and left at room temperature for 24 h to allow the sodium silicate to solidify. Once fully cured, the surface of the transparent bamboo sample was coated with a mixed solution of PFTS and TMCS in a 1:1 volume ratio using a dip-coating method. This coated sample was labeled as PFTS-TMCS@LSS-TB. The laboratory cost analysis for the transparent bamboo is presented in Table S6.

### Assembly of PSCs

The assembly of the PSCs and preparation of major components are based on the methods described in the literature [56,60]. Before the assembly, a 2-inch indium tin oxide (In_2_O_3_)_0.9_(Sn_2_O_3_)_0.1_ disk was employed as the target for Pulse Laser Deposition to coat transparent bamboo. As part of the assembly process, a TiO_x_ solution was spin-cast onto pre-cleaned ITO-coated transparent bamboo substrates. The coated substrates were dried at 150 °C for 30 min and then transferred to a glove box. Next, a perovskite solution was spread onto the TiO_x_ layer, followed by spin-coating at 1,000 rpm for 10 s and then at 4,000 rpm for 30 s with a ramp speed of 2,000 rpm/s. After 15 s into the second spin-coating step, 200 μl of anhydrous chlorobenzene, acting as an anti-solvent, was injected onto the film. The perovskite films were further annealed at 100 °C for 60 min on a hotplate. Subsequently, a Spiro-OMeTAD layer was spin-coated on top of the perovskite film. Finally, the fabrication of PSCs was completed by evaporating a gold electrode with a thickness of 100 nm at a rate of 0.03 nm s^−1^ using a shadow mask.

### Characterization

The microstructure of the samples was analyzed using a Zeiss Sigma300 SEM equipped with an EDX detector. Prior to observation, the samples were vacuum-sprayed with Au. FTIR spectra were acquired using a Thermo Scientific Nicolet iS5 spectrometer with a resolution of 4 cm^−1^ and a wavenumber range of 4,000 to 400 cm^−1^. XRD patterns were obtained using a Bruker D8 Advance TXS x-ray diffractometer with Cu Kα (target) radiation (*λ* = 1.5418 Å) at a scan rate (2*θ*) of 4° min^−1^, operating at 40 kV and 90 mA. Surface chemical compositions were analyzed using XPS with a Thermo Scientific K-Alpha instrument. The static contact angle was measured with a JY-82b Kruss DSA100 contact angle analyzer. Thermal stability was studied using a TGA5500 TG analyzer in an O_2_ environment, with a heating rate of 20 °C min^−1^. The transmission spectrum of the samples was measured using a Lambda 950 visible spectrophotometer, while the sample haze was assessed using a Diffusion EEL 57D haze meter from the UK. CONE tests were conducted following ISO 5660-2 guidelines, involving subjecting a sample with 100 mm (length) × 100 mm (width) × 3 mm (thickness) to a heat flux of 50 kW m^−2^ in a horizontal position. The test was performed in triplicate to ensure consistency.

### Determination of mechanical properties

The tensile properties of bamboo samples were tested using a universal testing machine (CMT 6502, Jinan Chenxin Testing Machine Manufacturing Co., Ltd., China) at a constant speed of 10 mm min^−1^. Tensile specimens were prepared according to the guidelines outlined in the Chinese National Standard GB/T 15780-1995 “Test Methods for Physical and Mechanical Properties of Bamboo” [61]. The dimensions of the sample were 160 mm × 10 mm × *t* mm (where “*t*” represents the radial thickness). The middle section of the specimen’s width was trimmed to 2 mm, similar to the shape of a dumbbell, with a gauge length of 60 mm. To ensure accuracy and reliability, the test was conducted in 6 replicates. The tensile strength (*σ*_t_) and tensile modulus of elasticity (tensile MOE, *E*_t_) were calculated using Eqs. 1 and 2.

$$\sigma_{t}=\frac{P_{\max}}{\mathrm{bt}}$$ (1)

where *P*_max_ is the failure load; *b* and *t* are the width and thickness of the middle part of dumbbell-shaped specimens, respectively.

$$E_{t}=\frac{∆\sigma }{∆\varepsilon }\;$$ (2)

where Δ*σ* represents the variation between the maximum and minimum stress values in the elastic region, and Δ*ε* denotes the difference between the maximum and minimum strain values within the elastic region.

The flexural properties of bamboo samples were measured using a universal mechanical testing machine (CMT 6502) equipped with a 3-point flexural device. For the flexural test, the dimensions of the sample used were 160 mm × 10 mm × *t* mm (where “*t*” represents the radial thickness) based on the Standard GB/T 15780-1995. The span for the flexural test was set at 120 mm, and the crosshead speed was maintained at 10 mm min^−1^. The test was performed in 6 replicates to ensure a consistent outcome. The bending strength (*σ*_b_) and bending modulus of elasticity (bending MOE, *E*_b_) were calculated by Eqs. 3 and 4.

$$\sigma_{b}=\frac{3P_{\max}L}{2bh^{2}}$$ (3)

where *L* is the span length; *b* and *h* are the width and height of specimen, respectively.

$$E_{b}=\frac{PL^{3}}{4bh^{3}f}$$ (4)

where *P* represents the variation between the maximum and minimum load experienced in the elastic region, and *f* represents the variation between the maximum displacement and minimum displacement within the same elastic region.

### Determination of photovoltaic properties

The photocurrent–voltage (*J*–*V*) measurement was conducted using an AM 1.5G solar simulator as the light source. The incident light intensity was calibrated at 100 mW·cm^−2^ using a standard Si solar cell. To obtain the *J*–*V* curves, the linear sweep voltammetry method was employed with a Keithley 2400 source-measure unit.

## Data Availability

The data that support the findings of this study are available within this paper and/or included in the Supplementary Materials and from the corresponding author upon request. Source data are provided with this paper.
