# Supplementary material for: A Novel Flame-Retardant, Smoke-Suppressing, and Superhydrophobic Transparent Bamboo
Source: Research (Wash D C). 2024 Feb 14;7:0317. doi: 10.34133/research.0317 (PMC10865110; doi:10.34133/research.0317)
Supplement: Supplementary 1 — Figs. S1 to S7 Tables S1 to S6 Movies S1 and S2 [file research.0317.f1.zip › Supporting Information.docx]

**Supporting Information**

**A novel** **flame-retardant,** **smoke-suppression, and** **superhydrophobic** **transparent bamboo**

Jiahui Su^1^†, Yadong Yang^1^†, Caichao Wan^1,2^†*, Xingong Li^1^, Yaling Chai^1^, Huayun Chai^1^, Jianzhong Yuan^1^, Yiqiang Wu^1^*

*^1^College of Materials Science and Engineering, Central South University of Forestry and Technology,* *Changsha 410004, P. R. China.*

*^2^Yihua Lifestyle Technology Co., Ltd, Huaidong Industrial Zone, Lianxia Town, Chenghai District, Shantou 515834, P. R. China.*

**Address correspondence to: wancaichaojy@163.com (C. Wan), wuyq0506@126.com (Y. Wu).*

†*These authors contributed equally to this work.*

**Table of contents**

**1. Key milestones in the history of glassmaking (Fig. S1)3**

**2. Possible reaction mechanism between PFTS-TMCS and LSS-TB (Fig. S2)4**

**3. Thermal stability of the natural bamboo and PFTS-TMCS@LSS-TB (Fig. S3)5**

**4. SEM image of PFTS-TMCS@LSS-TB after combustion (Fig. S4)6**

**5. Self-cleaning potential of transparent bamboo (Fig. S5)7**

**6.** **Tensile and flexural stress-strain curves of the natural bamboo and PFTS-TMCS@LSS-TB (Fig. S6)8**

**7. Tensile and flexural stress-strain curves of PFTS-TMCS@LSS-TB tested at ‒50 and 50 ºC (Fig. S7)9**

**8. FTIR characteristic bands and their assignments and sources (Table S1)10**

**9. CONE test parameters of the natural bamboo and PFTS-TMCS@LSS-TB (Table S2)11**

**10. Comparison of flame-retardant, smoke-suppression, and CO release properties between PFTS-TMCS@LSS-TB and congeneric transparent materials (Table S3)12**

**11. Photovoltaic performances of PSCs (Table S4)13**

**12. Tensile and flexural property parameters of the natural bamboo and PFTS-TMCS@LSS-TB (Table S5)14**

**13. Laboratory cost analysis of transparent bamboo (Table S6)15**

**14. Video S1: Combustion test for natural bamboo; Video S2: Combustion test for transparent bamboo** **16**

**15. References17**

1. **Key milestones in the history of glassmaking**


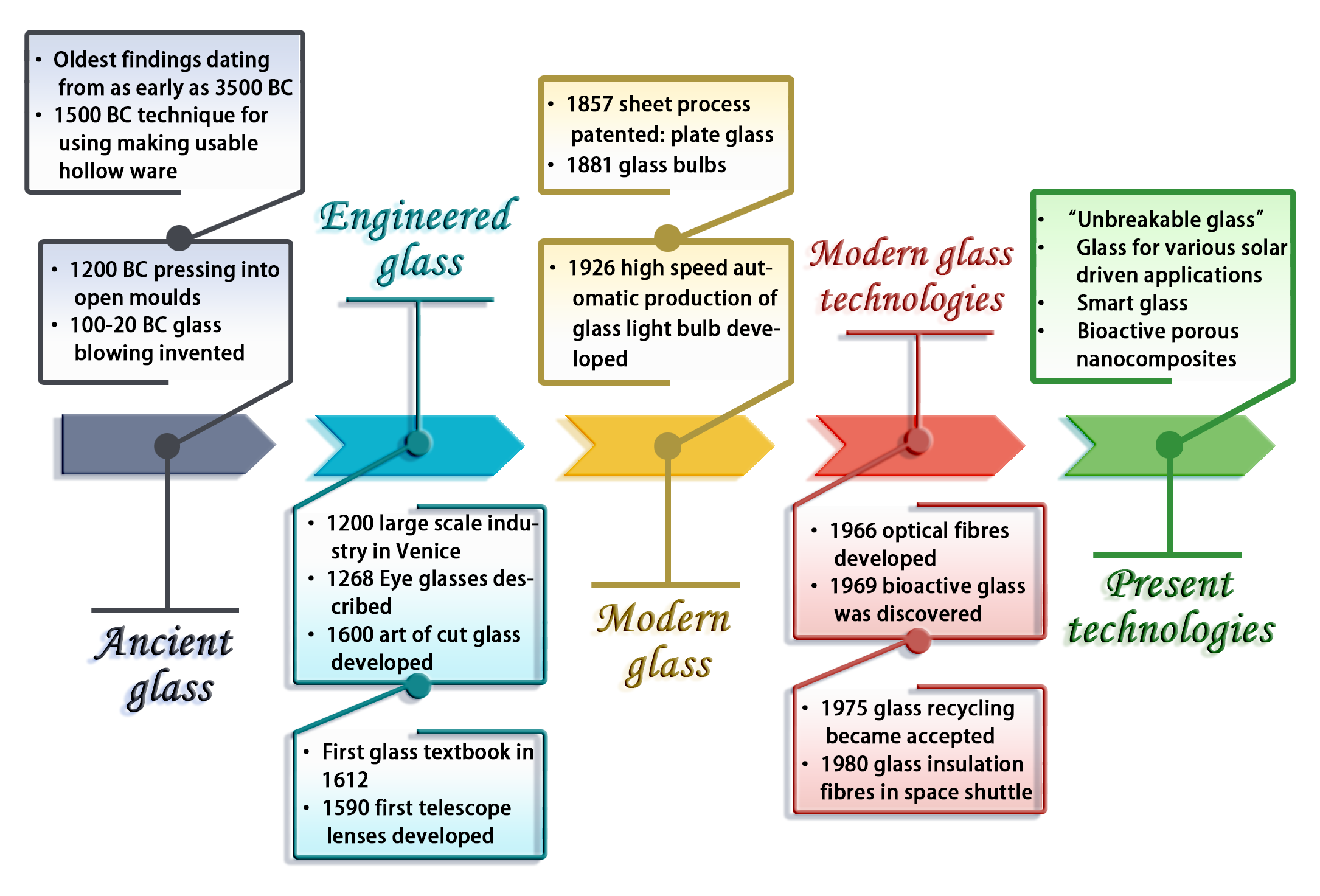


**Figure S1**. Key milestones in the history of glassmaking. (Drawn based on the literature [1]).

1. **Possible reaction mechanism between PFTS-TMCS and LSS-TB**


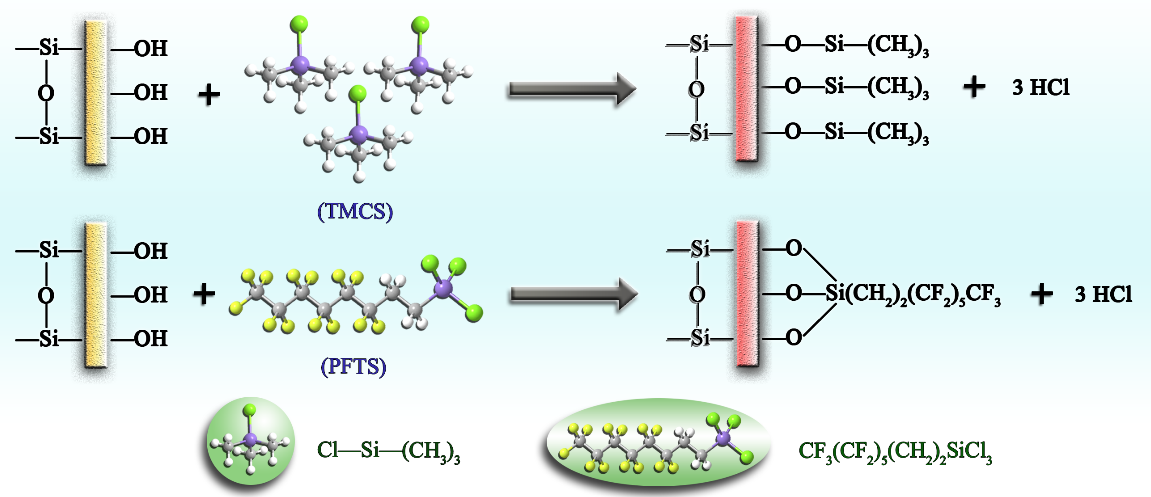


**Figure S2**. Schematic diagram of the possible reaction mechanism between PFTS-TMCS and LSS-TB.

1. **Thermal stability of the natural bamboo and PFTS-TMCS@LSS-TB**


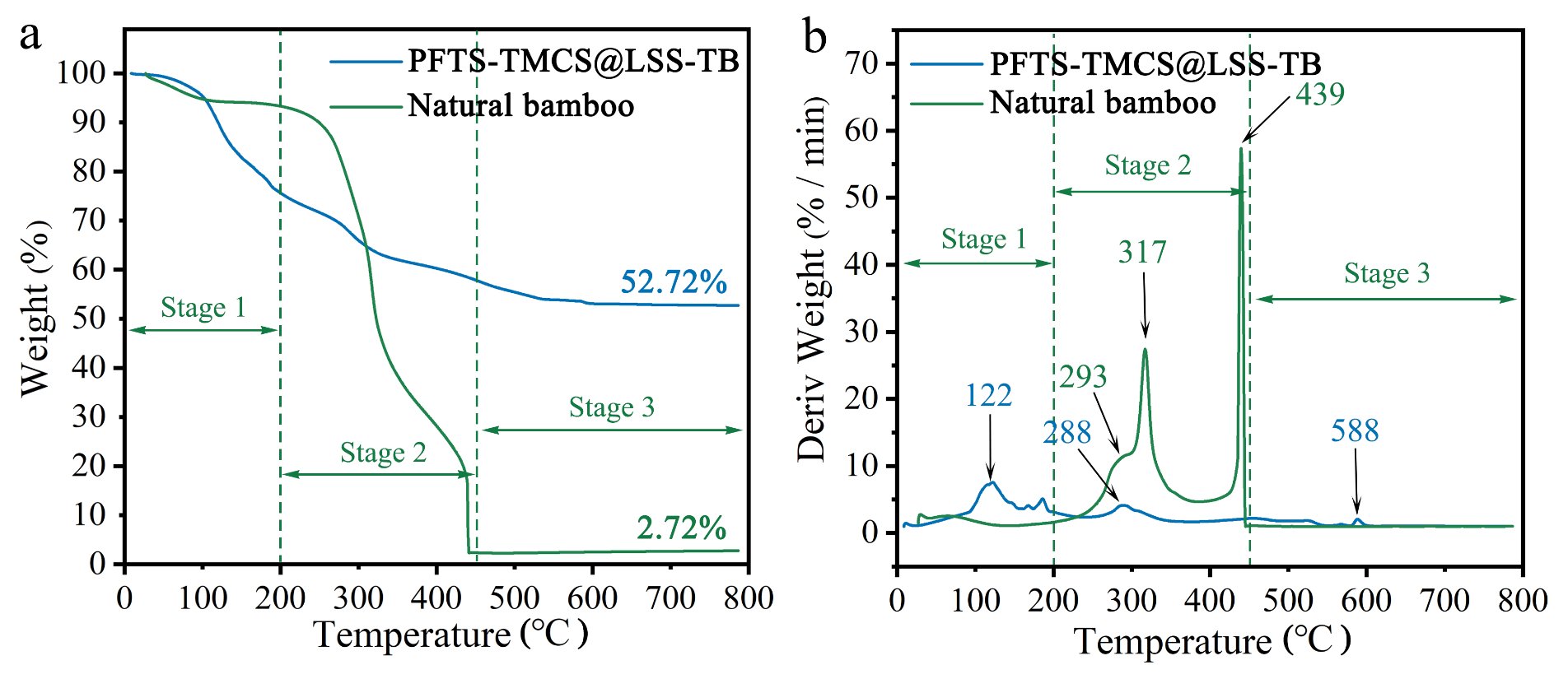


**Figure S3**. Thermal stability of the natural bamboo and PFTS-TMCS@LSS-TB. (a) TG curves. (b) DTG curves.

1. **SEM image of PFTS-TMCS@LSS-TB after combustion**


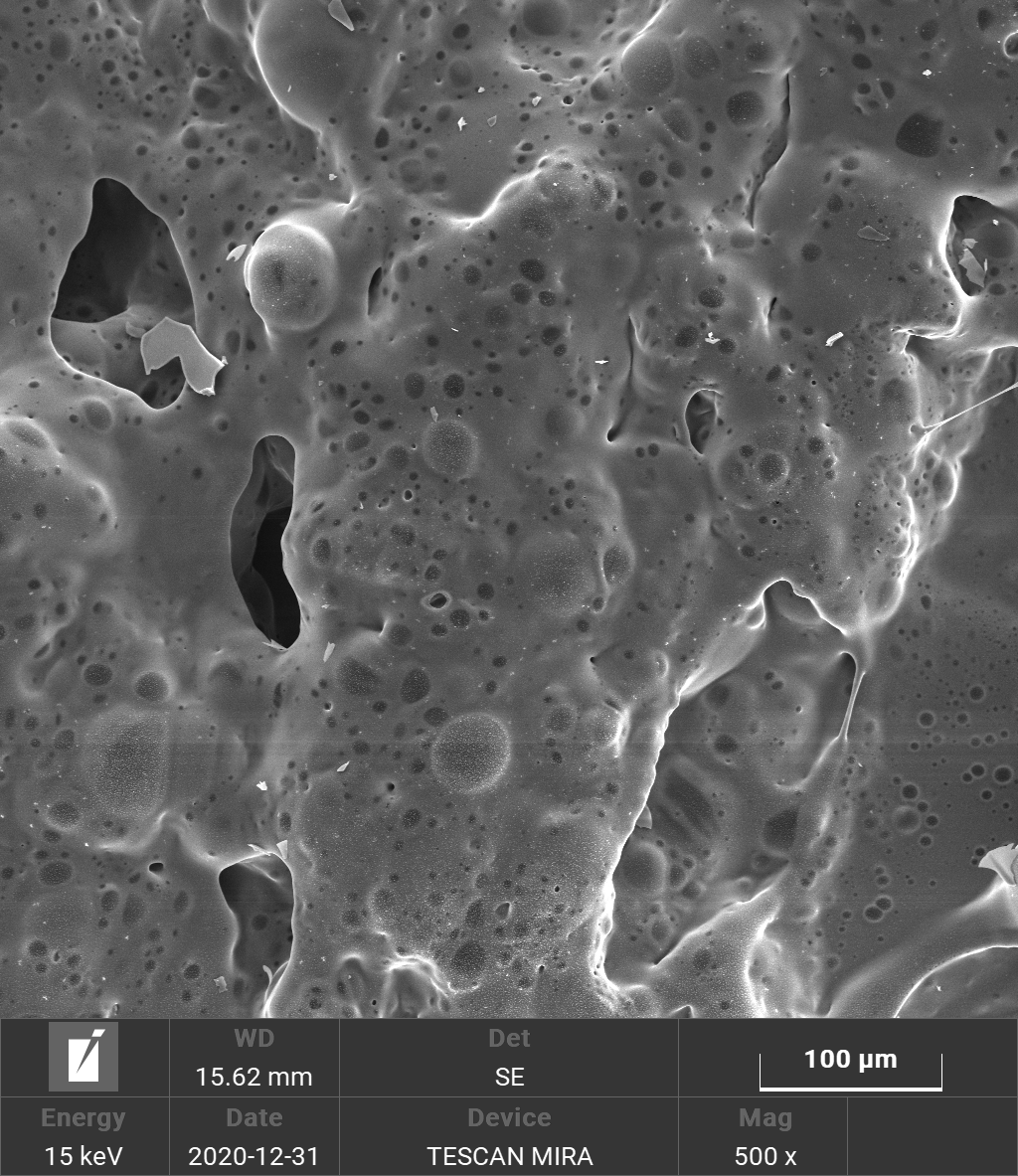


**Figure S4**. SEM image of PFTS-TMCS@LSS-TB after combustion.

1. **Self-cleaning potential of transparent bamboo**


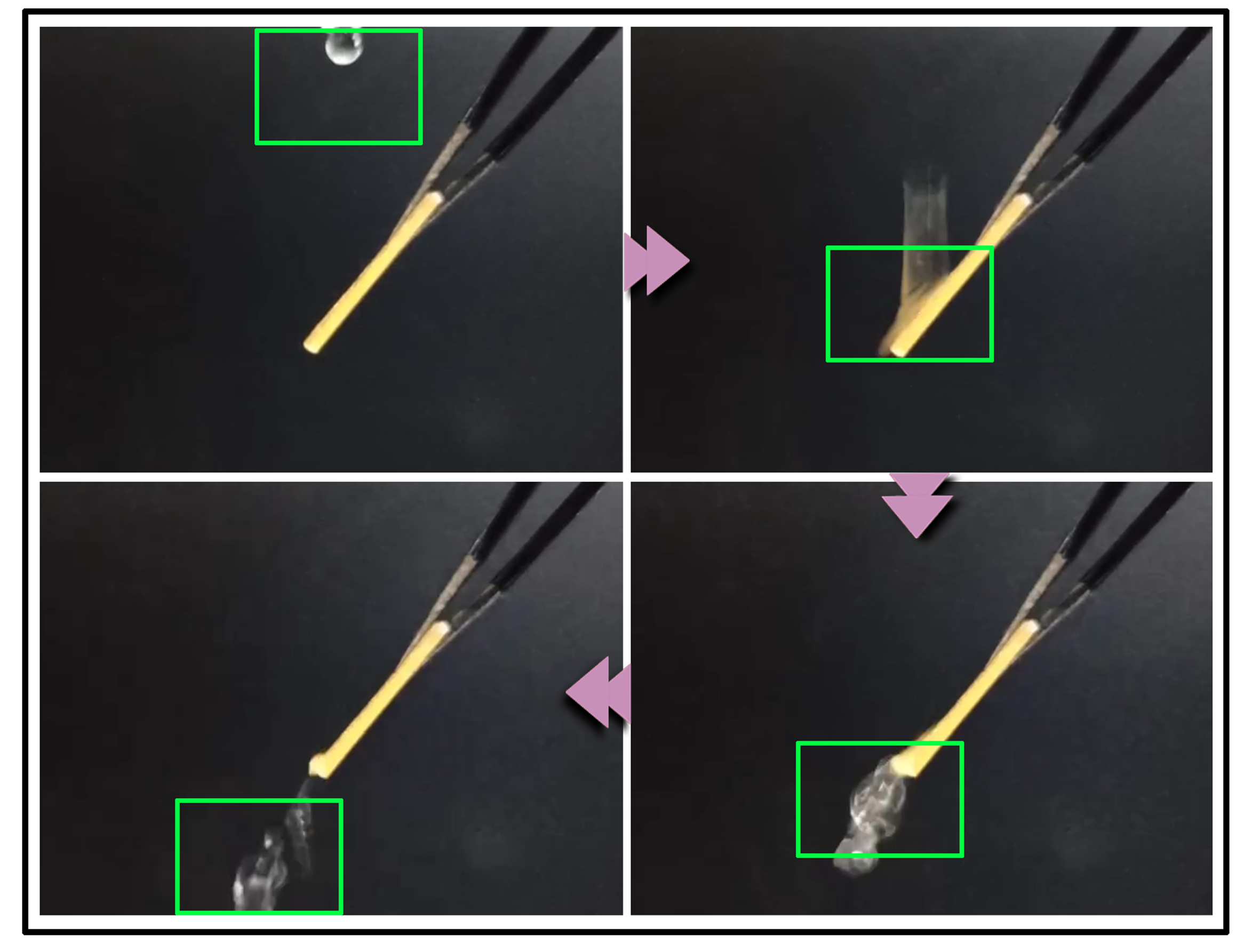


**Figure S5**. Illustrations demonstrating self-cleaning potential of transparent bamboo with water droplet gradually sliding off the surface.

1. **Tensile and flexural stress-strain curves of the natural bamboo and PFTS-TMCS@LSS-TB**


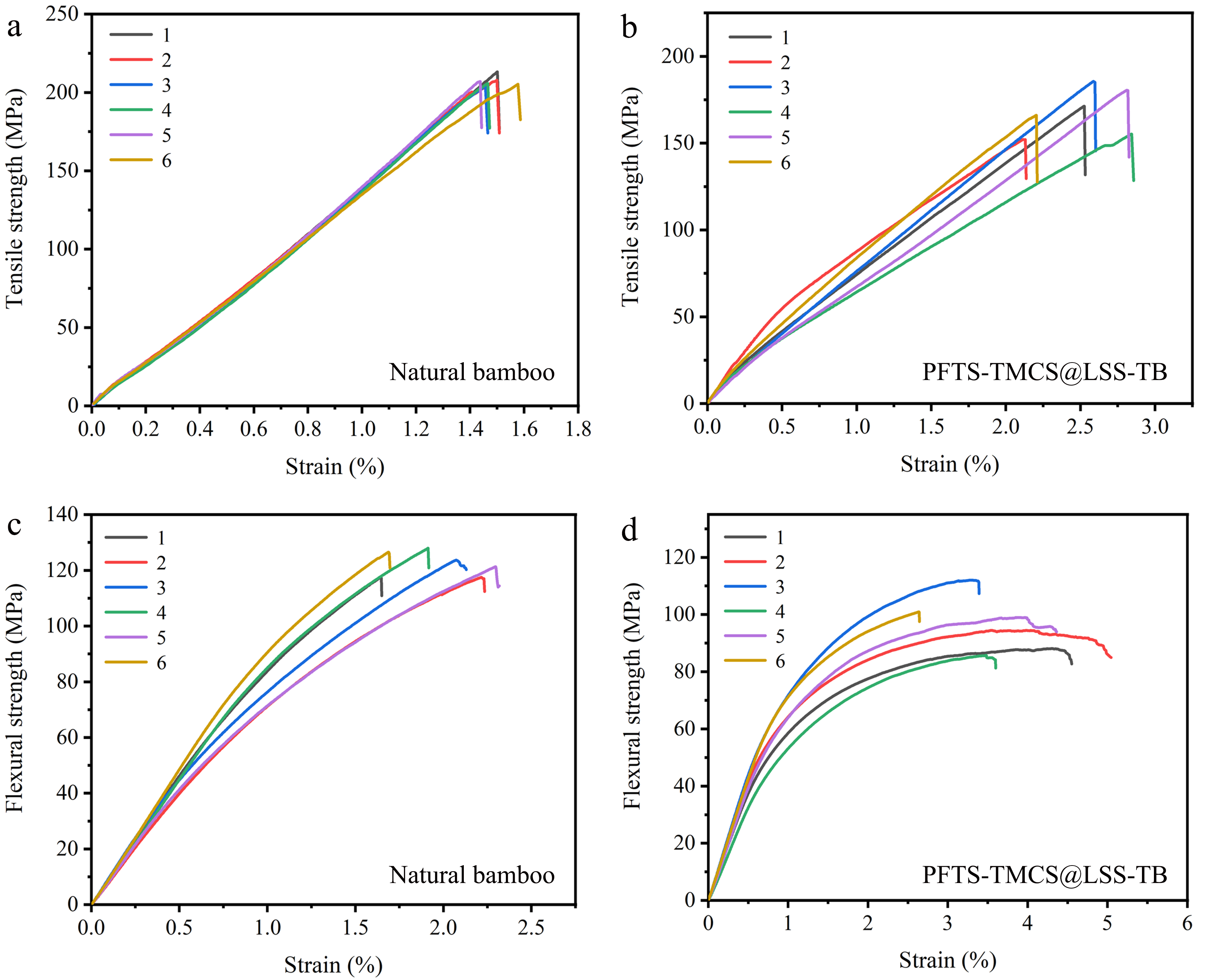


**Figure S6**. Tensile stress-strain curves of the natural bamboo (a) and PFTS-TMCS@LSS-TB (b). Flexural stress-strain curves of the natural bamboo (c) and PFTS-TMCS@LSS-TB (d). (Each test was repeated six times).

1. **Tensile and flexural stress-strain curves of PFTS-TMCS@LSS-TB tested at ‒50 and 50 ºC**


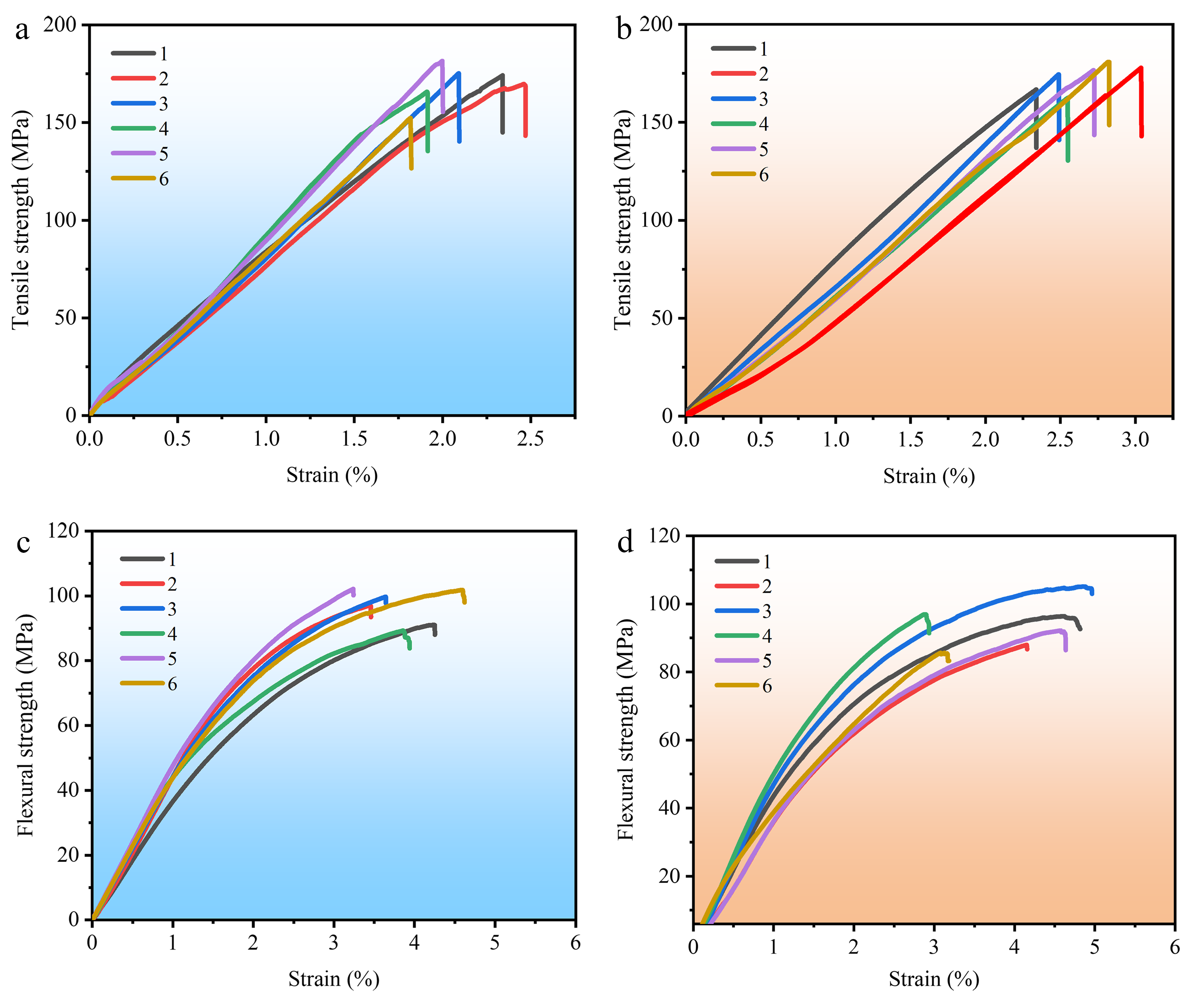


**Figure S7**. Tensile stress-strain curves of PFTS-TMCS@LSS-TB tested at (a) ‒50 ºC and (b) 50 ºC. Flexural stress-strain curves of PFTS-TMCS@LSS-TB tested at (c) ‒50 ºC and (d) 50 ºC. (Each test was repeated six times).

1. **FTIR characteristic bands and their assignments and sources**

**Table S1**. FTIR characteristic bands and their assignments and sources.

| **Absorption band (cm^‒1^)** | **Assignments** | **Sources** | **Samples** |
| --- | --- | --- | --- |
| 3332 | O‒H stretching | Cellulose, hemicellulose & lignin | Natural bamboo |
| 2898 | CH‒H stretching | Cellulose, hemicellulose & lignin | Natural bamboo |
| 1725 | C=O stretching in acetyl groups | Hemicellulose | Natural bamboo |
| 1619 | Aromatic skeleton C=C stretching | Lignin | Natural bamboo |
| 1518 | Aromatic skeleton C‒C stretching | Lignin | Natural bamboo |
| 1252 | Aromatic skeleton C‒O stretching | Lignin | Natural bamboo |
| 1370 | C‒H bending | Cellulose & hemicellulose | Natural bamboo |
| 1426 | (C_6_)‒CH_2_ bending | Cellulose | Natural bamboo |
| 1158 | C‒O‒C pyranose ring skeletal vibration | Cellulose | Natural bamboo |
| 1033 | C‒O‒C stretching | Cellulose | Natural bamboo |
| 896 | β-glycosidic linkages between the sugar units | Cellulose | Natural bamboo |
| 987 | Si‒O stretching | Liquid sodium silicate | LSS-TB |
| 1110 | Symmetrical shrinkage of Si‒O‒Si | Liquid sodium silicate | LSS-TB |
| 808 | Si‒(CH_3_)_3_ hydrophobic groups | PFTS-TMCS | PFTS-TMCS@LSS-TB |

1. **CONE test parameters of the natural bamboo and PFTS-TMCS@LSS-TB**

**Table S2**. CONE test parameters of the natural bamboo and PFTS-TMCS@LSS-TB.

| Samples | **Flame-retardant properties** | | | | | | **Smoke-suppression properties** | | | **CO & CO_2_ yields** | | | |
| --- | --- | --- | --- | --- | --- | --- | --- | --- | --- | --- | --- | --- | --- |
|  | TTI (s) | PHRR  (kW/m^2^) | MHRR  (kW/m^2^) | THR  (MJ/m^2^) | PEHC  (MJ/kg) | MEHC*^a^*  (MJ/kg) | PSPR  (m^2^ s^‒1^) | TSP  (m^2^) | MSEA  (m^2^/kg) | PCOY  (kg/kg) | MCOY*^b^*  (kg/kg) | PCO_2_Y  (kg/kg) | MCO_2_Y*^c^*  (kg/kg) |
| Natural bamboo | **20**  ± 1.6 | **289**  ± 11.3 | **73**  ± 2.5 | **13**  ± 0.8 | **27**  ± 2.1 | **14**  ± 1.1 | **0.026**  ± 0.002 | **1.0**  ± 0.04 | **110**  ± 2.9 | **0.013**  ± 0.0009 | **0.0045**  ± 0.0001 | **0.700**  ± 0.04 | **0.145**  ± 0.007 |
| PFTS-TMCS@LSS-TB | **116**  ± 3.5 | **13**  ± 1.2 | **3.9**  ± 0.21 | **0.7**  ± 0.04 | **13**  ± 0.4 | **1.7**  ± 0.16 | **0.001**  ± 0.00008 | **0.063**  ± 0.004 | **8.6**  ± 0.19 | **0.008**  ± 0.0001 | **0.0040**  ± 0.0001 | **0.076**  ± 0.006 | **0.058**  ± 0.002 |

*^a^*MEHC: mean EHC; *^b^*MCOY: mean CO yield; *^c^*MCO_2_Y: mean CO_2_ yield.

1. **Comparison of flame-retardant, smoke-suppression, and CO release properties between PFTS-TMCS@LSS-TB and congeneric transparent materials**

**Table S3**. Comparison of flame-retardant, smoke-suppression, and CO release properties between PFTS-TMCS@LSS-TB and congeneric transparent materials.

| **Samples** | **Flame-retardant properties** | | | **Smoke-suppression properties** | **CO yield** | **Ref.** |
| --- | --- | --- | --- | --- | --- | --- |
|  | TTI  (s) | PHRR  (kW/m^2^) | THR  (MJ/m^2^) | PSPR  (m^2^/s) | MCOY  (kg/kg) | ---- |
| EP | 59 | 1063.1 | 76.1 | 0.55 | 0.054 | [2] |
| HBD/EP | 93 | 528.5 | 35.9 | 0.24 | 0.093 | [2] |
| PMMA | 18 | 783 | 98 | 0.038 | 0.01 | [3] |
| MMA/HPD copolymers | 27 | 556 | 76 | 0.15 | 0.14 | [3] |
| DDM/EP | 88 | 817.9 | 61.1 | 0.2287 | 0.0347 | [4] |
| DDM/HPNFR/EP | 82 | 743.9 | 55.2 | 0.2445 | 0.0307 | [4] |
| PLA | 59 | 469 | 76.2 | 0.002 | 0.0067 | [5] |
| PLA/APP@CS | 57 | 406 | 69.4 | 0.007 | 0.0126 | [5] |
| PLA/APP@CS@Si | 57 | 387 | 66.6 | 0.005 | 0.0091 | [5] |
| DMDHEU | 17 | 400 | 36 | 0.027 | 0.007 | [6] |
| MGUP/BA | 22 | 380 | 20 | 0.003 | 0.004 | [6] |
| DMDHEU/MGUP/BA | 21 | 290 | 24 | 0.008 | 0.004 | [6] |
| PFTS-TMCS@LSS-TB | 116 ± 3.5 | 13 ± 1.2 | 0.7 ± 0.04 | 0.001 ± 0.00008 | 0.004 ± 0.0001 | This work |

1. **Photovoltaic performances of PSCs**

**Table S4**. Photovoltaic performances of PSCs assembled on the transparent bamboo or traditional glass.

|  | ***J*_SC_ (mA cm^-2^)** | ***V*_OC_ (V)** | ***J*_max_ (mA cm^-2^)** | ***V*_max_ (V)** | ***FF*** | ***η* (%)** |
| --- | --- | --- | --- | --- | --- | --- |
| PSCs assembled on the traditional glass | 20.85 | 1.12 | 19.22 | 0.82 | 0.675 | 15.76 |
| PSCs assembled on the transparent bamboo | 22.78 | 1.16 | 20.88 | 0.87 | 0.687 | 18.17 |

The fill factor (*FF*) and power conversion efficiency (*η*) of the assembled cells were calculated based on eqs. (1) and (2) [7]:

$FF\text{ }(\%)=\frac{J_{\text{max}}\times V_{\text{max}}}{J_{\text{sc}}\times V_{\text{oc}}}\times100$ (1)

$\eta\text{ }(\%)=\frac{J_{\text{max}}\times V_{\text{max}}}{P_{\text{in}}}\times100=\frac{FF\times V_{\text{oc}}\times J_{\text{sc}}}{P_{\text{in}}}\times100$ (2)

where *V*_OC_ is the open circuit voltage (V), *J*_SC_ is the short circuit current density (mA cm^‒2^), *P*_in_ is the power of incident light radiation, and *V*_max_ (V) and *J*_max_ (mA cm^‒2^) are the voltage and current density from the *J*‒*V* curves at the maximum point of power output, respectively.

1. **Tensile and flexural property parameters of the natural bamboo and PFTS-TMCS@LSS-TB**

**Table S5** Tensile and flexural properties of the natural bamboo and PFTS-TMCS@LSS-TB.

| Samples | Properties | Test no. 1 | Test no. 2 | Test no. 3 | Test no. 4 | Test no. 5 | Test no. 6 | Average | Standard deviation |
| --- | --- | --- | --- | --- | --- | --- | --- | --- | --- |
| Natural bamboo | Tensile strength (MPa) | 213.2 | 207.9 | 204.3 | 205.7 | 207.0 | 205.3 | 207.2 | 3.2 |
|  | Tensile modulus (GPa) | 13.2 | 13.0 | 13.2 | 12.9 | 13.9 | 13.0 | 13.2 | 0.4 |
|  | Elongation at break (%) | 1.5 | 1.5 | 1.5 | 1.5 | 1.4 | 1.6 | 1.5 | 0.06 |
|  | Toughness (MJ/m^3^) | 1.6 | 1.6 | 1.5 | 1.5 | 1.4 | 1.7 | 1.6 | 0.1 |
|  | Flexural strength (MPa) | 117.5 | 117.6 | 123.8 | 128.1 | 121.5 | 126.8 | 122.6 | 4.5 |
|  | Flexural modulus (GPa) | 9.3 | 10.7 | 9.8 | 9.0 | 8.7 | 9.9 | 9.6 | 0.7 |
|  | Fracture deflection (mm) | 10.1 | 10.7 | 10.4 | 11.3 | 11.0 | 11.6 | 10.9 | 0.6 |
| PFTS-TMCS@LSS-TB | Tensile strength (MPa) | 171.3 | 152.1 | 185.5 | 155.3 | 180.4 | 166.0 | 168.4 | 13.3 |
|  | Tensile modulus (GPa) | 6.7 | 8.7 | 7.1 | 5.4 | 6.0 | 6.3 | 6.7 | 1.1 |
|  | Elongation at break (%) | 2.5 | 2.1 | 2.6 | 2.8 | 2.8 | 2.2 | 2.5 | 0.3 |
|  | Toughness (MJ/m^3^) | 2.2 | 1.9 | 2.5 | 2.4 | 2.6 | 2.0 | 2.3 | 0.3 |
|  | Flexural strength (MPa) | 88.2 | 94.5 | 111.2 | 85.8 | 99.1 | 101.0 | 96.6 | 9.3 |
|  | Flexural modulus (GPa) | 6.2 | 8.6 | 9.1 | 6.7 | 6.5 | 8.7 | 7.6 | 1.3 |
|  | Fracture deflection (mm) | 29.9 | 36.7 | 22.6 | 23.7 | 31.3 | 24.6 | 28.1 | 5.5 |

1. **Laboratory cost analysis of transparent bamboo**

**Table S6**. Laboratory cost analysis of flame-retardant, smoke-suppression, and superhydrophobic transparent bamboo (dimensions: 5 cm × 5 cm × 0.3 cm).

| **Step 1: Delignification** | Bamboo chip | | Distilled water | Absolute ethanol^a^ | | NaClO_2_ solution^a^ | Glacial acetic acid^a^ | Electric charge |
| --- | --- | --- | --- | --- | --- | --- | --- | --- |
| Usage | A piece | | 60 mL | 60 mL | | 2 g NaClO_2_ + 50 mL water | < 1 mL | < 0.2 kW h |
| Price | 0.04 RMB | | 0.09 RMB | 0.22 RMB | | 0.19 RMB | < 0.08 RMB | < 0.17 RMB |
| **Step 2: Penetration of liquid sodium silicate** | Liquid sodium silicate | Electric charge | | | ╱ | ╱ | ╱ | ╱ |
| Usage | 20 g | | < 0.05 kW h | ╱ | | ╱ | ╱ | ╱ |
| Price | 0.07 RMB | | < 0.04 RMB | ╱ | | ╱ | ╱ | ╱ |
| **Step 3: Superhydrophobic modification** | PFTS^a^ | | TMCS^a^ | ╱ | | ╱ | ╱ | ╱ |
| Usage | 5 g | | 5 g | ╱ | | ╱ | ╱ | ╱ |
| Price | 0.21 RMB | | 0.27 RMB | ╱ | | ╱ | ╱ | ╱ |

^a^Chemicals including absolute ethanol, NaClO_2_ solution, glacial acetic acid, PFTS, and TMCS can be reused for treating multiple bamboo chips.

1. **Videos**

**Video S1**. Combustion test for natural bamboo.

**Video S2**. Combustion test for transparent bamboo.

Please find the videos in the attached file. Thank you!

**References**

[1] K. L. Edwards, E. Axinte, L. L. Tabacaru, A critical study of the emergence of glass and glassy metals as “green” materials. Materials & Design, 50 (2013) 713-723.

[2] S. Dai, X. Yu, R. Chen, H. Zhou, Z. Pan, Transparent epoxy resin material with excellent fire retardancy enabled by a P/N/S containing flame retardant, Journal of Applied Polymer Science, 138 (2021) 50263.

[3] W. Xie, B. Wang, Y. Liu, Q. Wang, Z. Yang, Flame retardancy of a novel high transparent poly(methyl methacrylate) modified with phosphorus-containing compound, Reactive and Functional Polymers, 153 (2020) 104631.

[4] X. Hu, H. Yang, Y. Jiang, H. He, H. Liu, H. Huang, C. Wan, Facile synthesis of a novel transparent hyperbranched phosphorous/nitrogen-containing flame retardant and its application in reducing the fire hazard of epoxy resin, Journal of Hazardous Materials, 379 (2019) 120793.

[5] L. Liu, M. Yao, H. Zhang, Y. Zhang, J. Feng, Z. Fang, P. Song, Aqueous self-assembly of bio-based flame retardants for fire-retardant, smoke-suppressive, and toughened polylactic acid, ACS Sustainable Chemistry & Engineering, 10 (2022) 16313-16323.

[6] T. Jiang, X. Feng, Q. Wang, Z. Xiao, F. Wang, Y. Xie, Fire performance of oak wood modified with N-methylol resin and methylolated guanylurea phosphate/boric acid-based fire retardant, Construction and Building Materials, 72 (2014) 1-6.

[7] J. Roncali, Luminescent solar collectors: quo vadis? Advanced Energy Materials, 10 (2020) 2001907.
